# Genetic diversity and population structure analysis of 418 tomato cultivars based on single nucleotide polymorphism markers

**DOI:** 10.3389/fpls.2024.1445734

**Published:** 2024-12-03

**Authors:** Weijie Xu, Chao Gong, Peiting Mai, Zhenxing Li, Baojuan Sun, Tao Li

**Affiliations:** Guangdong Key Laboratory for New Technology Research of Vegetables, Vegetable Research Institute, Guangdong Academy of Agricultural Sciences, Guangzhou, China

**Keywords:** genetic relationship, population structure, SNPs, DNA fingerprint, SNP markers

## Abstract

**Introduction:**

Tomato (*Solanum lycopersicum*) is a highly valuable fruit crop. However, due to the lack of scientific and accurate variety identification methods and unified national standards, production management is scattered and non-standard, resulting in mixed varieties. This poses considerable difficulties for the cataloging and preservation of germplasm resources as well as the identification, promotion, and application of new tomato varieties.

**Methods:**

To better understand the genetic diversity and population structure of representative tomato varieties, we collected 418 tomato varieties from the past 20 years and analyzed them using genome-wide single nucleotide polymorphism (SNP) markers. We initially assessed the population structure, genetic relationships, and genetic profiles of the 418 tomato germplasm resources utilizing simplified genome sequencing techniques. A total of 3,374,929 filtered SNPs were obtained and distributed across 12 chromosomes. Based on these SNP loci, the 418 tomatoes samples were divided into six subgroups.

**Results:**

The population structure and genetic relationships among existing tomato germplasm resources were determined using principal component analysis, population structure analysis, and phylogenetic tree analysis. Rigorous selection criteria identified 15 additional high-quality DNA fingerprints from 50 validated SNP loci, effectively enabling the identification of the 418 tomato varieties, which were successfully converted into KASP (Kompetitive Allele Specific PCR) markers.

**Discussion:**

This study represents the first comprehensive investigation assessing the diversity and population structure of a large collection of tomato varieties. Overall, it marks a considerable advancement in understanding the genetic makeup of tomato populations. The results broadened our understanding of the diversity, phylogeny, and population structure of tomato germplasm resources. Furthermore, this study provides a scientific basis and reference data for future analysis of genetic diversity, species identification, property rights disputes, and molecular breeding in tomatoes.

## 1 Introduction

Tomato (*Solanum lycopersicum*) is one of the most valuable vegetable crops worldwide. It is widely cultivated on all continents except Antarctica due to its good adaptability (Liu et al., 2023; Wang et al., 2023). However, modern tomato cultivars, similar to other crops, are facing problems caused by a narrowed genetic backgrounds. These issues are aggravated by climate change and frequent extreme weather events (Wang et al., 2023). Thus, it is necessary to collect and utilize more tomato germplasms to meet the future requirements of tomato breeding. Tomato landraces, as with most crops, are less inbred and have more genetic diversity than modern varieties. They have a long planting history and can adapt well to certain locations by harboring many biotic and abiotic stress-resistance genes. However, mistakes in naming landraces and duplications of genotypes have often occurred during the collection of landrace resources as landraces are typically maintained by local farmers over generations. Therefore, it is necessary to know the genetic backgrounds of these landraces for name corrections, duplicate removals, conservation, and tomato improvements. Although the genetic diversity of tomato landraces has previously been analyzed using simple sequence repeat (SSR) and SNP markers, numerous genotypes are still not well differentiated due to the lack of available markers or low polymorphism (Wang et al., 2023).

DNA molecular marker technology is extensively employed for the identification of plant varieties due to notable advantages such as independence from environmental conditions and species limitations, ease of operation, consistent and comprehensive locus identification, and considerable polymorphism (Yao et al., 2020; Liu, 2022). Owing to its unique advantages, molecular marker technology is widely applied in the research of forest plant species. In the field of plant biology, SSR and single nucleotide polymorphism (SNP) markers are utilized for the construction of fingerprint databases (Manivannan et al., 2021), the identification of genetic relationships, the development of genetic maps, and the isolation and cloning of diverse genes in various species including *Brassica oleracea* (Shen et al., 2021), *Triticum aestivum* (Li et al., 2018), *Camellia sinensis* (Karunarathna et al., 2021), and *Ipomoea batatas* (Wang et al., 2010). Previous research conducted genetic detection on 39 honeysuckle germplasm resources using eight pairs of high-quality SNP fluorescence markers (Liu, 2022). The development of SNP markers exhibits a higher abundance and greater degree of polymorphism. In addition, SNP markers are also easy to detect and statistically analyze, and can be identified using high-throughput automatic detection methods, which can greatly reduce the investment of manpower and material resources (Pei et al., 2015; Hata et al., 2023). SNPs display high-density characteristics and demonstrate remarkable stability within the genome. This facilitates their seamless integration into automated genotyping methods applicable to the entire genome (Gong et al., 2016; Xu et al., 2023; Yamaguchi et al., 2023). Numerous other high-throughput, low-cost SNP genotyping platforms have also been developed and widely utilized in important crop species such as *Triticum aestivum*, *Zea mays*, *Glycine max*, and *Vigna unguiculata* (Allen et al., 2011; Hiremath et al., 2012; Wang et al., 2021a; Wang et al., 2021b).

The KASP (Kompetitive Allele Specific PCR) system, developed by KBiosciences, exhibits superior characteristics compared to alternative methods for SNP genotyping. It boasts remarkable precision, excellent versatility in terms of target sites, and cost-effectiveness. Most importantly, it has been demonstrated as suitable for accurately identifying SNP sites across a notable quantity of samples (Yang et al., 2018). SNP markers are important in the analysis of crop genetic diversity and the establishment of linkage maps. They are also capable of achieving comprehensive high-throughput detection typing throughout the entire process (Song et al., 2015). At present, KASP has been applied to many species such as *Triticum aestivum*, *Malvaceae*, and *Oryza sativa* (Trick et al., 2012; Byers et al., 2012; Yang et al., 2019).

Wild tomatoes have adapted to a variety of ecological environments in Asia and western South America, including the Galapagos Islands. They can be found at altitudes ranging from near sea level to 3,600 m and display a wide range of genetic and phenotypic diversity (Peralta et al., 2008; Tieman et al., 2017; Tieman et al., 2017). SNP molecular markers provide many advantages in tomato breeding, including improving extensive marker variation, standardizing variation data, clear marker position delineation, and mature technical methods (Val et al., 2020; Liu, 2022). We employed the markers recommended for constructing DNA fingerprint databases in the International Union for the Protection of New Plant Varieties Biological and Molecular Marker Technology Molecular Testing Guidelines and the General Principles of DNA Fingerprinting Methods for Plant Variety Identification in China (NY/T 2594-2016) (Hayward et al., 2015; Fengge et al., 2018; Li et al., 2020). Therefore, it is crucial to effectively utilize the inherent diversity found in wild germplasm for the improvement of the genetic composition of tomatoes. To accomplish this objective, a thorough investigation into the extent of genetic diversity and population structure within tomato species is essential for genetic enhancement and preservation. However, previous research has raised concerns regarding the potential underestimation of tomato species diversity due to a lack of representativeness or a limited number of samples. There is an urgent need to investigate the molecular diversity present in tomatoes to enhance their productivity and quality. In our study, we analyzed the population composition, genetic affiliations, and genetic profiles of the 418 tomato germplasm resources from several regions worldwide. This was achieved using a simplified method of genome sequencing technology. High-quality DNA fingerprints were extracted from the validated 50 SNP loci and were confirmed to effectively identify the 418 tomato varieties using KASP platforms. These fingerprints have been gradually employed for the authentication of new cultivars, seed purity, and variety genuineness. We then analyzed the population structure and identified varieties within the tomato germplasm collection. The findings can significantly enhance future investigations in the fields of genetic diversity, species identification, property rights conflicts, and molecular breeding. This can be achieved by providing a scholarly foundation and benchmark data for these endeavors.

## 2 Materials and methods

### 2.1 DNA extraction and library construction

The tomato cultivar (*Solanum pimpinellifolium* (PIM), *Solanum lycopersicum* var. cerasiforme (CER), and *Solanum lycopersicum* (BIG)) was utilized to construct full-length DNA libraries extracted from leaves, as per previous research (Gramazio et al., 2020). Plants were grown in the field according to a randomized complete block design in Zhongluotan (23°23 N, 113°26 E), Guangzhou City, Guangdong Province, China. Each line consisted of three replicated blocks. After grinding the leaf samples, DNA was extracted using an RNeasy Kit (Qiagen) following the manufacturer’s instructions. Following purification and DNA digestion with RNase-free DNase (Qiagen), DNA quality was assessed using a NanoDrop ND-2000 (A260/A280 1.9–2.1) and an Agilent 2100 Bioanalyzer (28S/18S 1.8–2.0). A total of 3 µg of DNA per sample was utilized for the library construction on the Illumina HiSeq 4000 platform as per the manufacturer’s instructions and index codes. Library construction and paired-end sequencing were subsequently performed (Hanania et al., 2004; Trapnell et al., 2014).

### 2.2 Simplified genome sequencing and data analysis

Library construction and paired-end sequencing were performed by Beijing Biomarker Technologies. Subsequently, data quality control was conducted on the original sequenced reads (Giovannoni, 2004; Dellaporta et al., 2016). The Burrows–Wheeler Aligner (BWA, v.0.7.17-R1188) was utilized to align the sequenced reads with the reference genome (Byers et al., 2012). The reference tomato genome was the ‘Heinz 1706’ genome (*S. lycopersicum*) (https://solgenomics.net/ftp/genomes/Solanum_lycopersicum/Heinz1706/assembly/build_4.00/), and the parameters were configured as -M -R. The insert size and coverage depth of each sample were assessed, and variations were identified by comparing the alignment positions of clean reads on the reference genome (Sato et al., 2012). The files generated from the comparison were then converted to bam format using Samtools (v.1.9; https://samtools.sourceforge.net/). Following this, Picard MarkDuplicates (v.2.21.2; https://www.ncbi.nlm.nih.gov/geo/query/acc.cgi?acc=GSM5970534) was utilized to identify and remove duplicate tags, ensuring that only high-quality reads were retained for subsequent analysis.

### 2.3 Discovery and genotyping of SNP

To ensure the acquisition of high-quality SNPs, we employed a unique that included mapped paired-end reads for the SNP calling process. The genotype likelihood parameter of the genomic site for each individual was calculated using SOAPsnp (https://sourceforge.net/projects/soapsnp/) with default parameters (Gu et al., 2013). To validate the accuracy of our SNP calling results, we compared them with SNP data from a previous study involving 120 individuals and focusing on 10 candidate genes obtained through genome re-sequencing (Du et al., 2014). The SNP data in the previous study was determined using PCR-Sanger sequencing. The relative accuracy rate of our SNP calling exceeded 99%, demonstrating the high quality and reliability of our platform for SNP calling.

### 2.4 Genetic diversity and population structure analysis

Based on the high-quality SNPs obtained, we utilized VCFtools v.0.1.16 (https://vcftools.sourceforge.net/) to conduct principal component analysis (PCA) and other population divergence statistics (Petr et al., 2011; Abdelhafez et al., 2019) with default parameters. Subsequently, an evolutionary tree of the 418 tomato samples was constructed using the maximum likelihood (ML) method in FastTree (v.2.1.9). The genetic structure of the population was analyzed using PLINK1.9 (Sorkheh et al., 2007; Chang et al., 2015). PLINK1.9 and VCFtools64 v.0.1.16 were used to perform PCA and other population divergence statistics, including nucleotide diversity and genetic differentiation (FST).

### 2.5 Phylogenome analysis

The high-quality SNPs identified above underwent a second round of filtering to enhance the accuracy and efficiency of phylogenetic analysis as per previous research (Zhang et al., 2021). Phylogenomic analysis was conducted by inferring orthologs and orthogroups using OrthoFinder (v.2.5.4) with the default settings and an activation of ‘-M msa’ (Emms and Kelly, 2018). The longest predicted protein of each individual gene was used as the representative input for the OrthoFinder analysis. TrimAl (v.1.4.12) was used to remove poorly aligned regions of protein multiple sequence alignments (Capella-Gutiérrez et al., 2009). RAxML (v.8.2.12) was employed to construct ML phylogenetic trees using the GAMMAJTT model (Stamatakis, 2014; Silvestro et al., 2012).

### 2.6 GO enrichment analysis

Candidate genes were analyzed using the AmiGO Term Enrichment tool (http://amigo.geneontology.org/) for GO analysis. The R (R Core Team) module CLUSTERPROFILER, available at Bioconductor (http://bioconductor.org), was utilized to identify enriched GO terms associated with candidate genes. We applied multiple testing corrections using the Benjamini and Hochberg FDR method (Green et al., 2007). GO terms with a corrected P < 0.05 were deemed significantly enriched.

### 2.7 Fingerprint construction and data analysis

The construction of DNA fingerprinting is achieved by utilizing high-quality SNPs (Xu et al., 2017; Obayes et al., 2024). To achieve simplicity, efficiency, and cost-effectiveness, we aimed to utilize the minimum number of markers possible, allowing us to identify the maximum number of varieties (Wang et al., 2009). Based on the size and frequency distribution of polymorphic information content (PIC) values, we meticulously selected 50 core markers of SNP sites with a high detection rate and considerable polymorphism. The obtained SNP loci were screened based on the following criteria: (1) they were evenly distributed across the different chromosomes; (2) no genotype data was missing; (3) the number of unmeasured materials at the site was <20 and the minor allele frequency (MAF) was ≥0.34; (4) the PIC was >0.35; (5) the Hardy–Weinberg equilibrium (HWE)-tested p-value was ≥0.01; and (6) there were no other mutations within 100 bp before and after the polymorphic site. These markers had the capability to distinguish all varieties and a DNA fingerprint was subsequently constructed. Furthermore, the 15 most efficient SNP combinations were further screened to identify tomato varieties with reduced costs and faster growth rates (Hiremath et al., 2012; Jones and Mackay, 2015).

### 2.8 Development and analysis of KASP markers

For each SNP site retained during the screening process, the surrounding sequences on both sides of the SNP were trimmed to a size of 100 bp. The KASP primers utilized in this study were developed and designed by LGC Genomics LLC (Beverly, MA, USA). Here, we used Primer 5.0 (https://primer-premier-5.software.informer.com/) to develop PCR primers for the competitive allele-specific PCR-single nucleotide polymorphism (KASP-SNP) linked markers. PCR was performed on parental lines to denote the polymorphic efficacy of the designed primers. Based on this preliminary PCR analysis, the polymorphic markers were used to screen the bulks and backcross population. The sequences of primers are listed in **Supplementary Table S1**.

## 3 Results

### 3.1 Genome sequencing and alignment to the tomato reference genome

**Figure 1:**
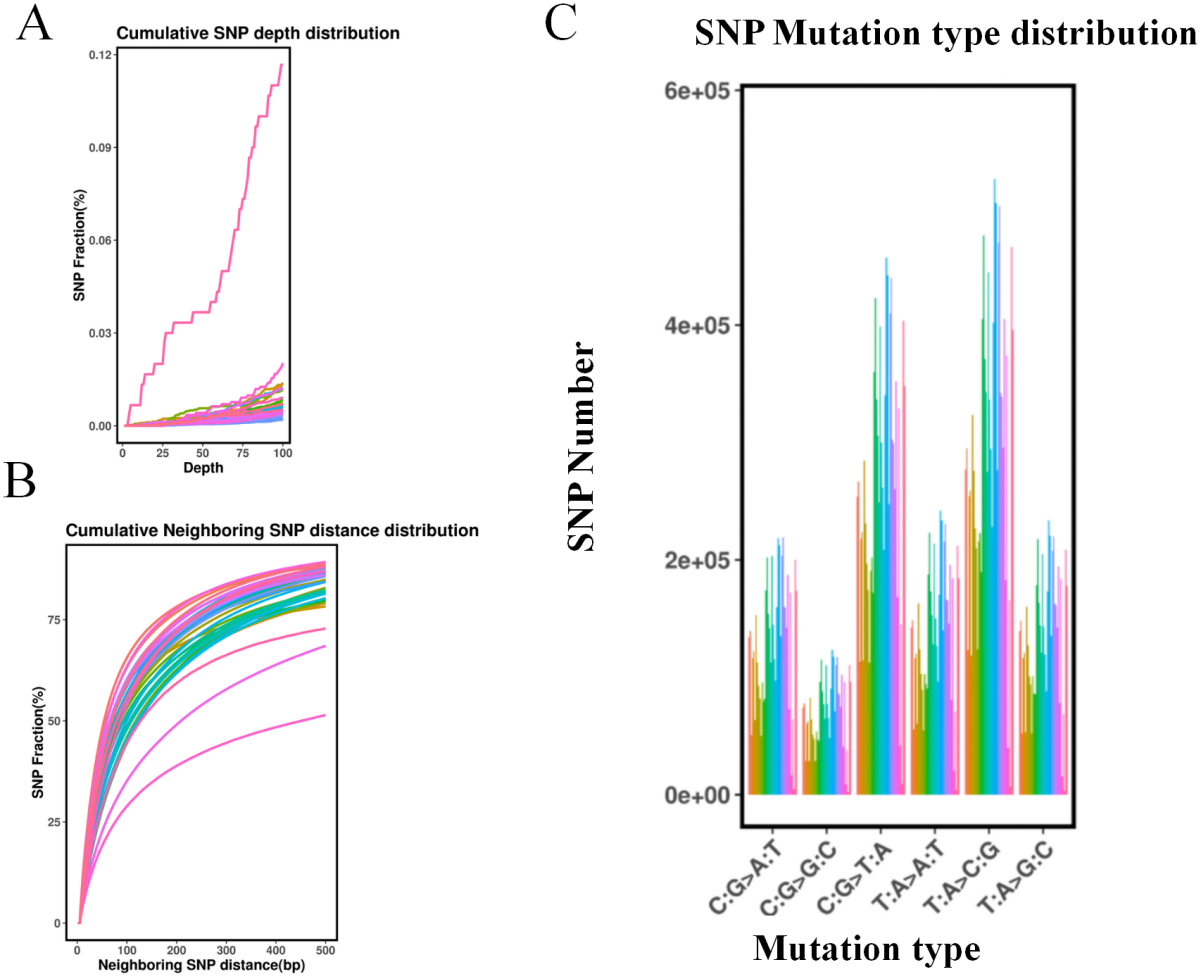
Single nucleotide polymorphism (SNP) type and distribution identification of 418 samples. **(A)** Cumulative SNP depth distribution. The horizontal axis represents the depth, and the vertical axis represents the fraction of SNPs. **(B)** Cumulative neighboring SNP distance distribution. The horizontal axis represents the neighboring SNP distance(bp) and the vertical axis represents the fraction of SNPs. **(C)** SNP mutation type distribution on each chromosome. The horizontal axis represents the mutation type, and the vertical axis represents the SNP number.

First, we constructed high-quality DNA fingerprints of 418 tomato samples from around the world from the past 20 years using high-quality SNPs (**Supplementary Figure S1**). The acquisition of this high-quality, clean data provides a robust foundation for our work (**Supplementary Table S1**). The original raw Illumina reads obtained through GBS (genotyping by sequencing) underwent quality control and data filtering to produce high-quality clean data, which serves as the foundation of this study. To obtain a high-quality genome for the 418 tomatoes, the sequencing of the tomato samples on the Illumina NovaSeq 6000 PE150 yielded a total of 196,612,427 PE reads (55.44 Gb) of clean data (**Figures 1A, B** and **Supplementary Table S1**). This is consistent with the estimated genome size obtained from the Illumina short read sequencing data. The average coverage depth of the loci reached 492.87, and the obtained clean reads were mapped to the ‘Heinz 1706’ reference genome with a mapping efficiency of 93.25%. The genotype deletion rate was determined as 4.71% (**Supplementary Table S1**). This assembly also demonstrated high quality compared to other organisms with sequenced reference genomes in previous studies (https://www.biorxiv.org/content/10.1101/2021.05.04.441887v1).

### 3.2 Identification and annotation of high-quality SNPs in the 418 tomato samples

**Figure 2:**
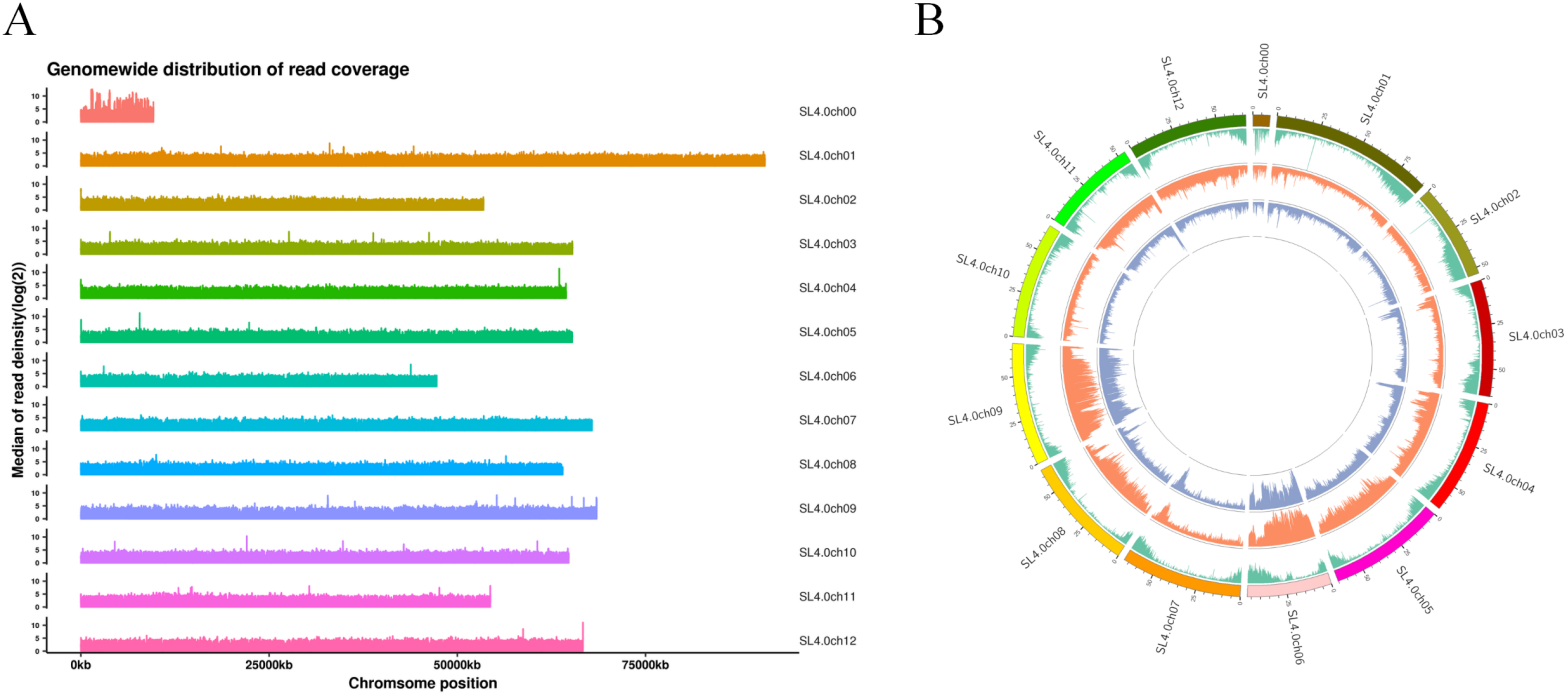
Single nucleotide polymorphism (SNP) type and distribution identification of 418 samples. **(A)** Number of SNPs on each chromosome. The horizontal axis represents the chromosome number, and the vertical axis represents the number of SNPs. **(B)** SNP density distribution on each chromosome. The horizontal axis represents the chromosome length, and the vertical axis represents the chromosome number. Different colors represent the number of SNPs in different regions.

After completing the sequencing, the Genome Analysis Toolkit (GATK; https://gatk.broadinstitute.org/hc/en-us) was used to identify a notable number of SNP variants in the 418 tomato samples. The identified SNPs were filtered, resulting in a total of 13,143,268 SNPs generated during the sequencing process (**Figure 2**). Furthermore, a total of 3,374,929 filtered SNPs were obtained and distributed across 12 chromosomes in the tomato genome (**Figure 2B**). Notably, the distribution of SNPs on each chromosome was depicted based on the number and density of SNPs. There were approximately 400,000 SNPs on chromosome 4 and 600,000 SNPs on chromosome 10 (**Figures 2A, B**). Chromosomes 3–9 exhibited a similar number of SNPs, with approximately 300,000 SNPs on each chromosome.

The analysis of these SNP-predicted mutation types revealed that, out of the six possible single base mutations, C/T and A/G transitions were the most prevalent, accounting for 26.43% and 28.51% of the total, respectively. Further analysis of the distribution of the SNPs in the genome revealed that 70.29% were located in intergenic regions, 6.85% were located in introns, 8.59% were located in the 1-kb region downstream of the transcription termination site, 0.05% were simultaneously located in the 1-kb region upstream of one gene and in the 1-kb region downstream of another gene, and 0.03 and 0.02% were located in the 5′ and 3′ UTRs (**Supplementary Figure S2**). **Figure 1C** presents the mutation type distribution of the SNPs in the genomic, where T:A replaces C:G, which is the most common type of variation. A total of 94,457 non-synonymous single nucleotide variants (SNVs) were predicted to cause changes in coding amino acids, while 62,439 synonymous SNVs were predicted not to alter amino acid sequences. The ratio of non-synonymous SNVs to synonymous SNVs was 1.51.

**Figure 3:**
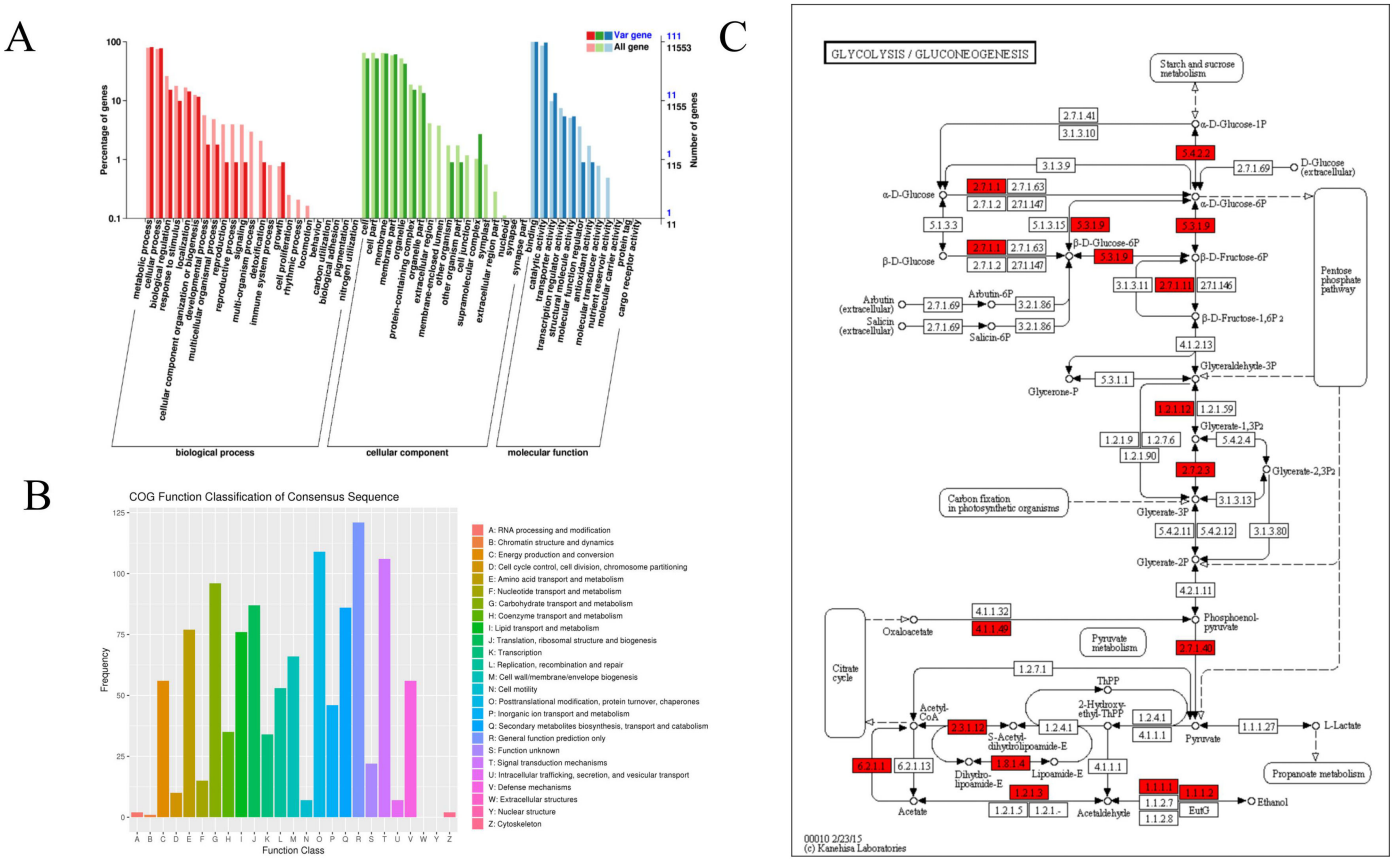
Functional annotation of single nucleotide polymorphisms (SNPs) in 418 samples. **(A)** GO annotation clustering of candidate mutated genes. Note: The horizontal axis represents the classification content of GO, the left side of the vertical axis represents the percentage of genes, and the right side represents the number of genes. **(B)** Classification diagram of mutated gene COG annotation. The horizontal axis represents the classification content of COG, and the vertical axis represents the number of genes. **(C)** Partial variant gene pathway metabolic map of GLYCOLYSIS/GLUCONEOGENESIS pathway. The numbers in the box represent the enzyme number, indicating that the corresponding gene is associated with this enzyme. The entire pathway is formed by many different enzymes undergoing complex biochemical reactions, and the mutated genes related to this pathway are marked in red boxes.

By employing a range of strategies, such as *de novo* prediction, homology-based methods, and transcriptome alignment, we identified and annotated genes. A total of 6,001 protein-coding genes were predicted in SNP, with 98.8% of these predicted genes exhibiting homologues in at least one of the six functional protein databases. Furthermore, a total of 5,910 genes were distributed across different chromosomes, representing approximately 90.14% of the total predicted genes. Among them, 28.4% of SNP sites caused changes in amino acids, including 1,678 candidate genes found in our study. To further understand the potential impact of mutation types between different varieties on the phenotype, the GO annotation of 1,678 candidate genes for several enriched biological pathways, including “RNA processing and modification,” “RNA processing and modification,” “basal transcription factor,” “starch and sucrose regulatory metabolism,” “ubiquitin-mediated proteolysis,” “terpenoid backbone biosynthesis,” “pentose and glucoronateinter conversions,” “glycolipid metabolism,” “flavonoid biosynthesis,” “fatty acid biosynthesis,” “elongation and degradation,” and “ascorbate and aldarate metabolism” (**Figure 3**). This suggests that a potential mechanism to overcome deleterious mutations occurred in important genes related to basic biological functions. For example, the *SRC2* gene encodes an activator of a calcium-dependent pathway that mediates reactive oxygen species production in response to cold stress, indicating the adaptation of various samples to different selections, climates, and environments.

### 3.3 Genetic relationships and population structure analysis

**Figure 4:**
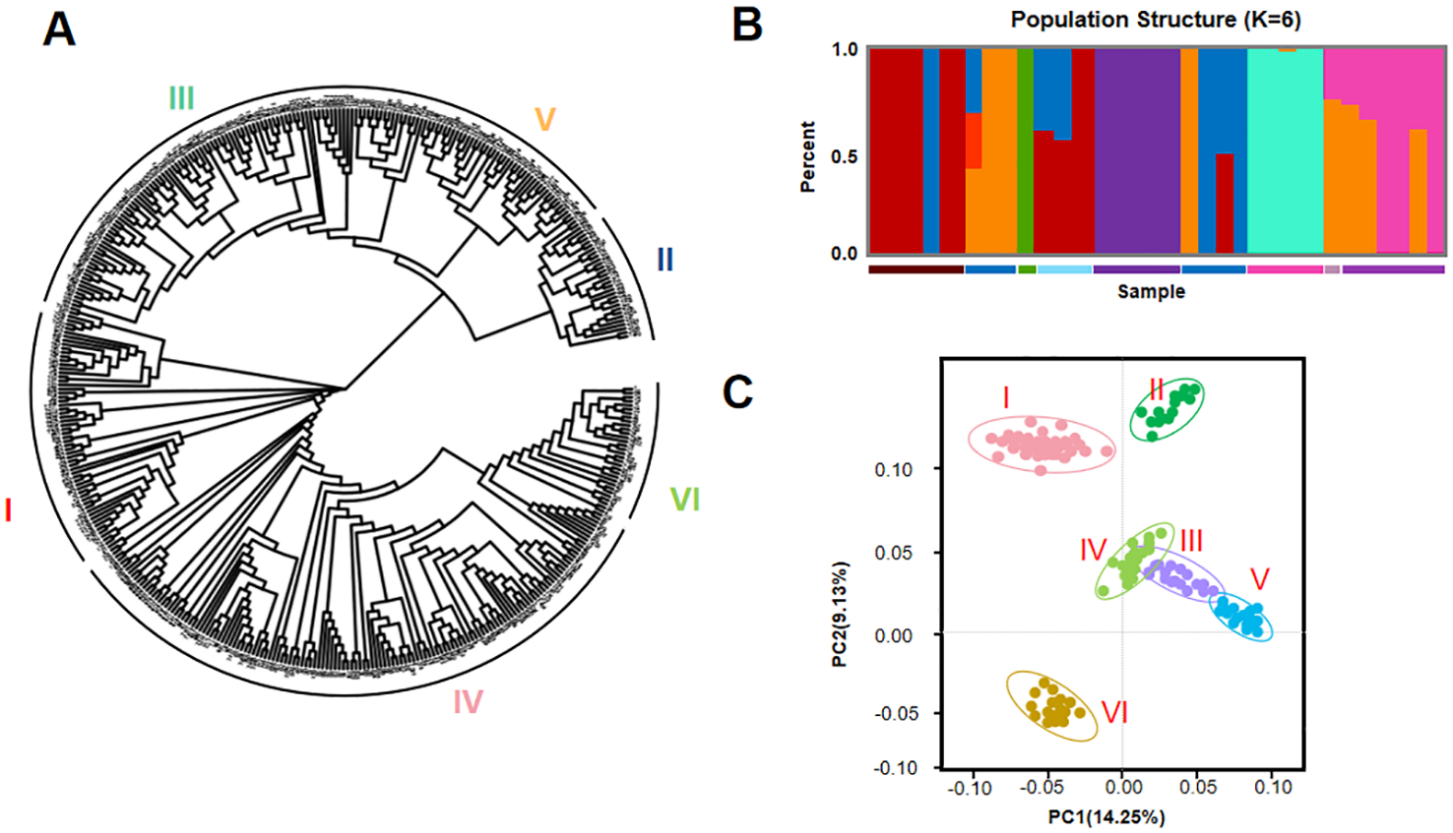
Phylogenetic analysis analysis of 418 cherry tomato varieties based on single nucleotide polymorphisms (SNPs). **(A)** Phylogenetic tree of 418 cherry tomato varieties. **(B)** Population structure of 418 cherry tomato samples when K = 6. **(C)** A two-dimensional diagram of principal component analysis (PCA) of 418 cherry tomato samples.

The phylogenetic tree showed that the 418 tomato varieties clustered into six groups, denoted as clades I, II, III, IV, V, and VI (**Figures 4A, B**). This classification is consistent with the variations in the five morphological traits of the row-type and adherence of the hulls. Each clade contained 39–128 members. Interestingly, three clades (I, III, and VI) were present in all continents, with high supporting values. In clade II, only 39 varieties were observed, suggesting this clade was lost during transition, selection, and breeding. Notably, we observed an extensive diversity of clade IV with most varieties. PCA was also utilized on the sequences of the high-quality SNPs identified in the 418 tomato accessions. The two-dimensional graph in **Figure 4** illustrates the values of each sample in the first (PC1) and second (PC2) principal components. The values in parentheses on the axis labels indicate the percentage of the total variation explained by PC1 and PC2, which were 14.25% and 9.13%, respectively. The geographical origins of the varieties and the PCA results jointly reveal that each of the four groups contained tomato accessions originating from different geographic regions (**Figure 3**). This study demonstrates that there is no considerable correlation between the PCA results and the geographical origin of the germplasm. In addition, we employed Plink and Frappe to analyze the population structure of the 418 tomato accessions. The determination of the number of clusters is typically based on the cross-validation error rate, with the optimal number identified as that with the lowest cross-validation error rate. As illustrated in **Figure 3**, the cross-validation error rate is minimized when K = 6, indicating that the 418 tomato accessions can be classified into six clusters. This is consistent with the phylogenetic analysis results. In addition, the test materials were not fully categorized based on their geographical origins, which aligns with the classification derived from the PCA. The neighbor-joining method in Tree Best was utilized to construct a phylogenetic tree for the 418 tomato accessions. The first three axes of the PCA further confirmed this population structure but showed more divergence between subgroups I and VI (**Figure 4C**). The remaining accessions did not cluster solely based on geographical origin. The other clades contained tomato accessions from different geographical regions, and those originating from the same region were distributed across all five clades. However, note that while these tomato accessions from the same source are complex and diverse in terms of genetic backgrounds, their clustering within the clade indicates interrelatedness and independence among them.

### 3.4 Design of KASP primers and selection of core SNP sites

SNP sites containing other mutations within 100 bp before and after the SNP were excluded. This resulted in a total of 737 remaining sites in the 418 tomato varieties. The flanking sequences of these sites were then extracted from the corresponding scaffold and aligned with the tomato reference genome at the chromosome level. Only the sites located on the chromosomes were retained, leading to a final selection of 71 SNP sites for the subsequent experiment. The 715 SNP sites demonstrated considerable polymorphism and a high degree of variety discrimination. The MAF and PIC values for these sites ranged from 0.346 to 0.500, and 0.350 to 0.375, with average values of 0.45 and 0.37, respectively. Notably, the percentage of PIC values falling within the range of 0.370–0.375 was found to be at 70.6%, indicating a strong level of polymorphism for these markers. The observed heterozygosity for the 715 variable SNP sites ranged from 0 to 0.64, with an average of 0.10. The genetic diversity within the germplasm collection was assessed and found to range from 0.45 to 0.50, with an average of 0.49. The combined MAF, PIC, and observed heterozygosity values were utilized for further screening of the SNP sites distributed across the 12 chromosomes. These sites did not have missing genotypic data, were predominantly homozygous variant sites, and were detected in as many individuals as possible. This screening yielded a total of 163 SNP sites. Subsequently, KASP primers were designed for these identified SNP loci, with successful conversion into KASP markers achieved for all 73 primer sets. The KASP markers were then typed and verified using GBS-genotyped tomato accessions (n = 418). Based on the typing results, we identified 46 KASP markers that demonstrate high accuracy (**Supplementary Figure S3**). These markers were selected as candidate markers for screening verification populations. Among them, those with superior performance were designated as core markers and utilized as candidate core primers for further analysis.

### 3.5 Fingerprint construction and verification of varieties

**Figure 5:**
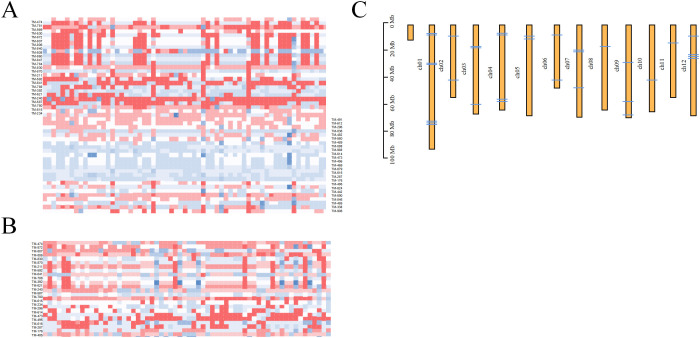
DNA fingerprinting of 418 tomato samples. **(A)** DNA fingerprint is composed of 50 single nucleotide polymorphisms (SNPs). **(B)** DNA fingerprint composed of 15 SNPs. Homozygous genotypes C/C, A/A, T/T, and G/G are represented by white, light red, crimson and blue. **(C)** Distribution of core markers on tomato chromosomes.

Based on the simplified sequencing results, SNP loci with a PIC value greater than 0.30—which were uniformly distributed on different chromosomes—were selected as the core loci for constructing the fingerprint of the tomato varieties in our study (**Figure 5**). Finally, we screened 50 high-quality SNP loci and constructed a fingerprint, successfully distinguishing the 418 tomato varieties. Among the 50 core SNPs, the PIC values for site markers 8, 17, 23, and 32 were maximized at 0.38, while the PIC values for Marker 4 were minimized at 0.30. The average PIC value was calculated as 0.34, indicating moderate polymorphism. Paired comparisons were conducted on the 418 tomato samples using this developed core SNP locus combination. Statistical analysis reveals considerable differences in the number of different loci among the 418 tomato samples, and different loci were observed in each pair of samples, demonstrating the high reliability of this locus combination. This indicates a substantial diversity in the genetic markers present within the different samples, highlighting the complexity and richness of the genetic information being studied. This study aimed to efficiently and cost-effectively differentiate tomato varieties. We focused on identifying the minimum number of SNPs required to distinguish 418 tomato varieties from the 50 core loci obtained. As a result, we obtained the simplest SNP combination, which is evenly distributed on 12 chromosomes (**Figure 5**). When utilizing a combination of 15 SNPs for sample differentiation, we determined at least one SNP variation between the two samples. This allows for effective discrimination among the 418 tomato varieties. Note that certain tomato samples could only be differentiated based on a single SNP. For example, “C20F31” and “C20F02” could only be distinguished by Marker 7, while “003” and “065” (SPL004) could only be distinguished by Marker 6. These results also indicate that the development of fingerprint maps is of great significance in the complex and chaotic genetic relationship background of tomato germplasm.

## 4 Discussion

### 4.1 Analysis of SNP-based genetic relationships among the 418 tomato germplasm reveals considerable polymorphism

Much horticultural research has been conducted on tomatoes due to their ease of cultivation, large number of plants, and wide planting area (Dargiri et al., 2021). In addition, a comprehensive overview of the variations in medicinal history, sources, characteristics, chemical composition, and other aspects of tomatoes has been provided (Tieman et al., 2017). However, although a wide variety of tomato cultivars have been extensively grown in previous studies, there is a lack of standardized and decentralized management. Further investigation is necessary to elucidate the genetic relationships and population structure of existing tomato varieties.

In this study, 418 tomato germplasm resources from around the world were collected and grouped into six subgroups using simplified sequencing, PCA, population structure analysis, and evolutionary tree construction. The results show similar outcomes. Subgroup IV comprises tomato cultivars that demonstrate high yields, robust resistance to pests and diseases, and desirable traits currently valued in the market. These findings emphasize the complex nature of wild tomato species lineages, in contrast to the relatively simpler lineages observed in systematically bred tomatoes (Liu, 2022). In the population structure analysis, all individuals displayed ancestral lines from the four subgroups. However, the genetic relationship between “C20F02” and “C20F07” was found to be direct and derived from a single pedigree. This discovery effectively explains their placement within subgroup IV on the evolutionary tree. Furthermore, four additional hybrids were included in this study. All hybrids inherited genetic traits from their parents, which aligns largely with expectations. Currently, our research group is conducting a more comprehensive analysis of hybrid characteristics.

Many genes previously reported to be involved in climate adaptation were identified here with variants to be associated with environmental variables, a significant functional enrichment could be detected in our study, the GO annotation of 1678 candidate genes about several enriched biological pathways were significantly shown in’basal transcription factor’, ‘starch and sucrose regulatory metabolism’, ‘ubiquitin-mediated proteolysis’, ‘terpenoid backbone biosynthesis’, ‘pentose and glucoronateinter conversions’, ‘fatty acid biosynthesis’, ‘elongation and degradation’, as well as ‘ascorbate and aldarate metabolism’. The variants of these genes also related to precipitation showed similar geographic distribution in frequencies. For example, this transcription factor with a lateral organ boundary (LOB) domain plays an essential role in root development in response to flooding and drought stresses. The SRC2 gene encodes an activator of a calcium-dependent pathway that mediates reactive oxygen species production in response to cold stress.

### 4.2 Efficient and convenient SNP fingerprints for the identification of the 418 tomato germplasm resources

Unlike the majority of previous studies that concentrate on tomato morphology and quality assessments (Zhang et al., 2011; Beckles et al., 2012; Li et al., 2022), the current study aimed to analyze the population structure of tomatoes, explore the differences among tomato varieties, and utilize SNP analysis to distinguish between different tomato varieties. This study not only provides a clear explanation of the population relationships among the existing 418 tomato germplasm resources but also offers further insights into the genetic relationships between different tomato varieties. These findings serve as valuable references and a foundation for the management of tomato germplasm resources, holding considerable importance for the future breeding of new tomato varieties. This research contributes to a comprehensive understanding of the genetic diversity within tomato germplasm resources and provides essential knowledge for their effective utilization in breeding programs. The implications of these findings are far-reaching, with the potential to considerably impact the development of improved tomato varieties with desirable traits (Muir et al., 2014). In our study, we utilized SNP molecular marker technology and DNA fingerprinting to obtain 196,612,427 SNPs from the 418 tomato samples. Among these, 50 core SNPs demonstrated strong identification ability for tomato germplasm. Furthermore, specific DNA fingerprinting analysis was conducted and developed. The fingerprint map, which was created using 50 core SNP markers, effectively distinguishes between different species and varieties of tomatoes. This study also identified 15 SNPs as the most compact combination of sites and the DNA fingerprints constructed based on the loci of these 15 SNP loci were utilized for the detection of various tomato samples. At least one distinct locus was identified in each variety of tomato, allowing for the rapid, accurate, and cost-effective differentiation of different species and varieties (Elbasyoni et al., 2018). Given the highly complex genetic background of tomato germplasm, this study conducted further research on varieties that exhibited several different loci in the fingerprints of the 15 SNP loci. After further analysis, it was found that these varieties showed high repeatability in DNA fingerprints and similarity in origin, species, and characteristics. Thus, when the DNA fingerprint difference between two varieties is ≤1 locus, the varieties share similarities in species, origin, parentage, and flower color. This indicates that the two varieties are likely to be either identical or highly similar.

### 4.3 Reliability and stability of the 15 tomato SNP core fingerprints

To evaluate the reliability of the 15 SNP loci DNA fingerprints, four samples were randomly selected from the initial 418 germplasm resources. PCR and Sanger sequencing were used for the analysis. The results of the SNP verification were consistent with previous findings, demonstrating reliability and stability with previous research (Liu, 2022). A remarkably high genetic stability was observed among these samples, with a consistency rate of 97.33%. This level of stability is consistent with the typical characteristics exhibited by tomatoes. However, it is important to note that the genotypes of samples “C20F31,” “C20F19,” “C20F15,” and “022,” underwent a change from A/A to A/G in Marker 10. This result may be attributed to the presence of a second allele of the SNP. In summary, the validation results of the 15 SNP loci and the genetic stability of tomato were as expected. The analysis confirmed the authenticity and reliability of the DNA fingerprint composed of these loci, and also verified the high genetic stability of tomato. This provides valuable data and theoretical support for tomato germplasm identification and SNP fingerprint methods.

This research provides new insights into the genetic diversity of tomato varieties, domestically and internationally, over the past 20 years. It also reveals important information about the genetic background of domestic tomato varieties. The findings of this study have major implications for basic research in tomato breeding, the protection of new plant varieties (PVPs), and genetic diversity.

## Data Availability

Raw data are available at the BIGD Genome Sequence Archive (https://bigd.big.ac.cn) under accession number CRA007031.
