# Supplementary material for: Genetic diversity and population structure analysis of 418 tomato cultivars based on single nucleotide polymorphism markers
Source: Front Plant Sci. 2024 Dec 3;15:1445734. doi: 10.3389/fpls.2024.1445734 (PMC11649422; doi:10.3389/fpls.2024.1445734)
Supplement: Supplementary file 1 [file DataSheet1.docx]

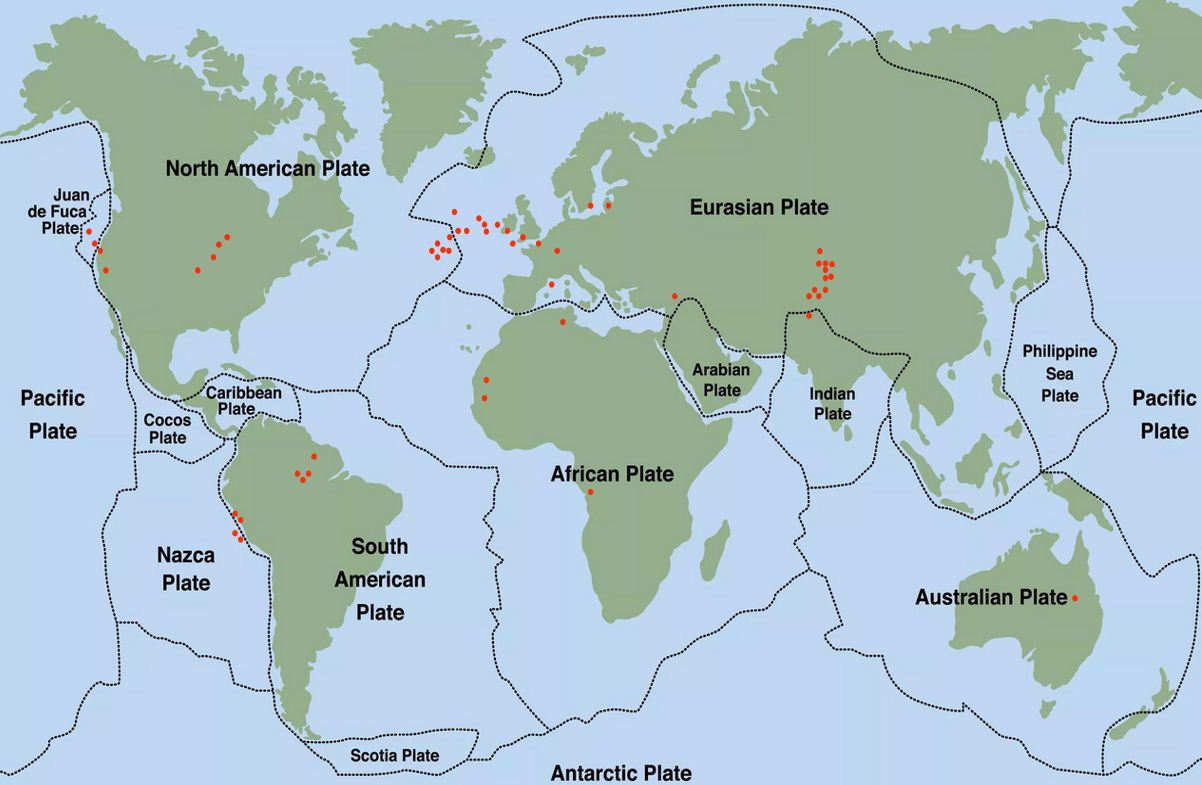


**Figure S1.** Distribution map of 418 tomato varieties all over around the world.


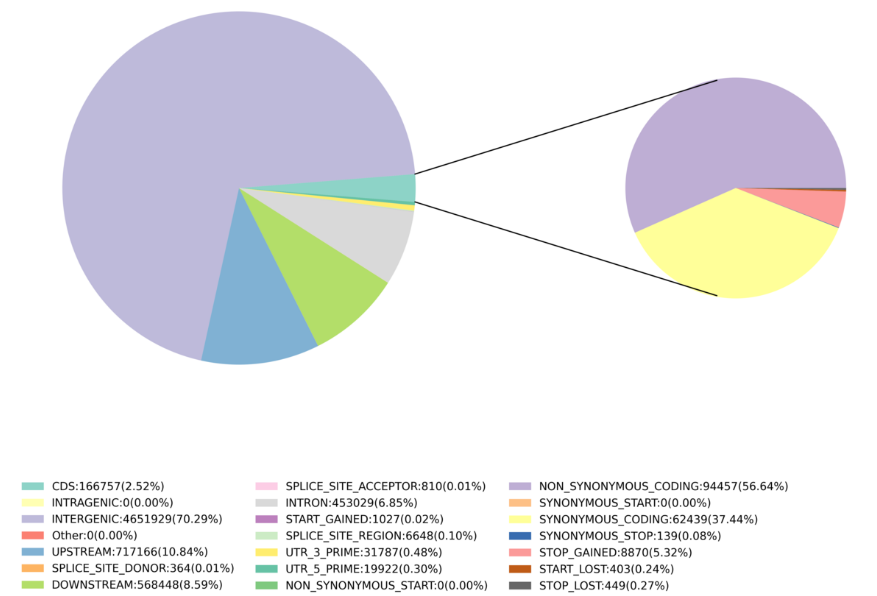


**Figure S2.** SNP mutation type dietribution and annotation in 418 cherry tomatoes.

**
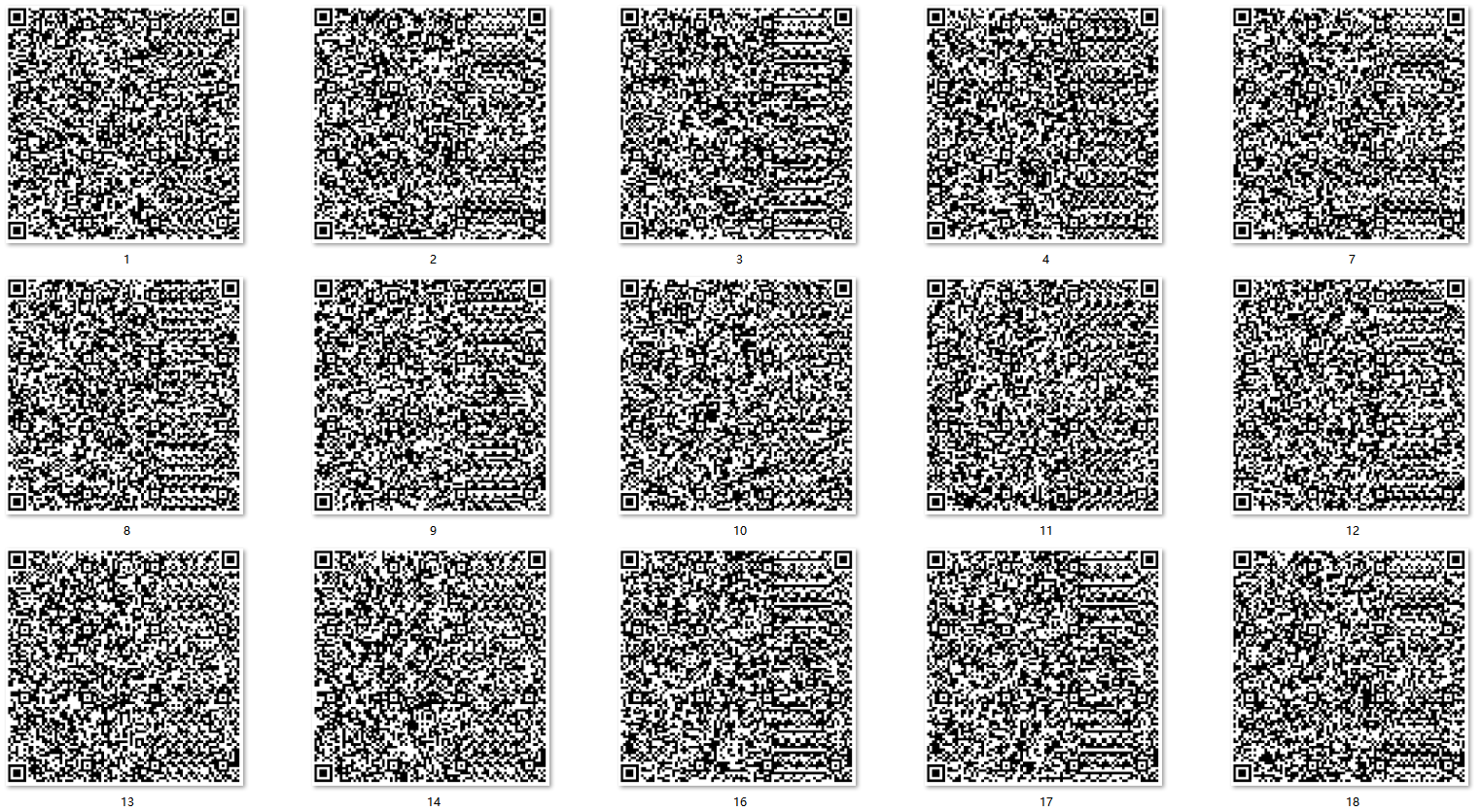
**

**
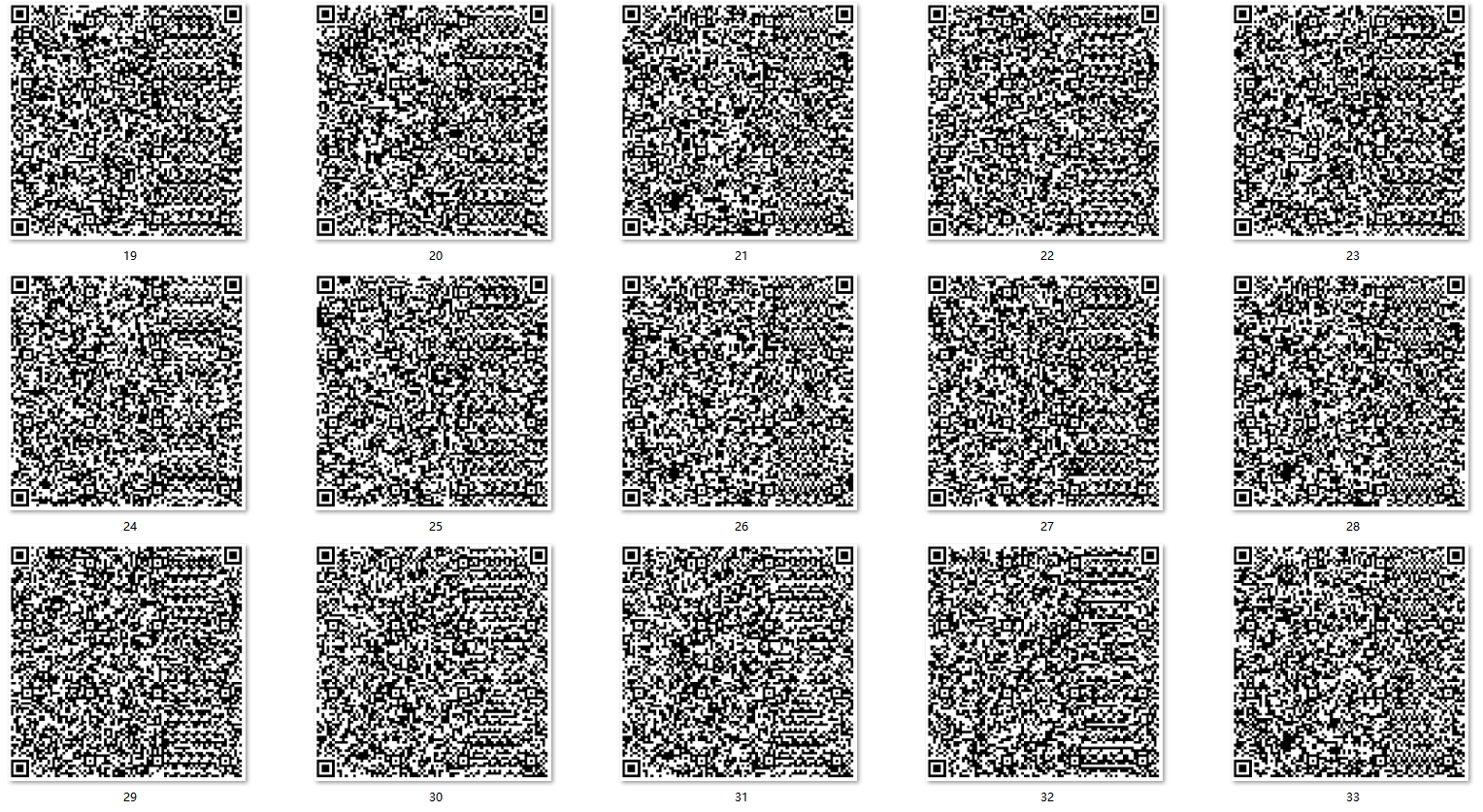
**

**
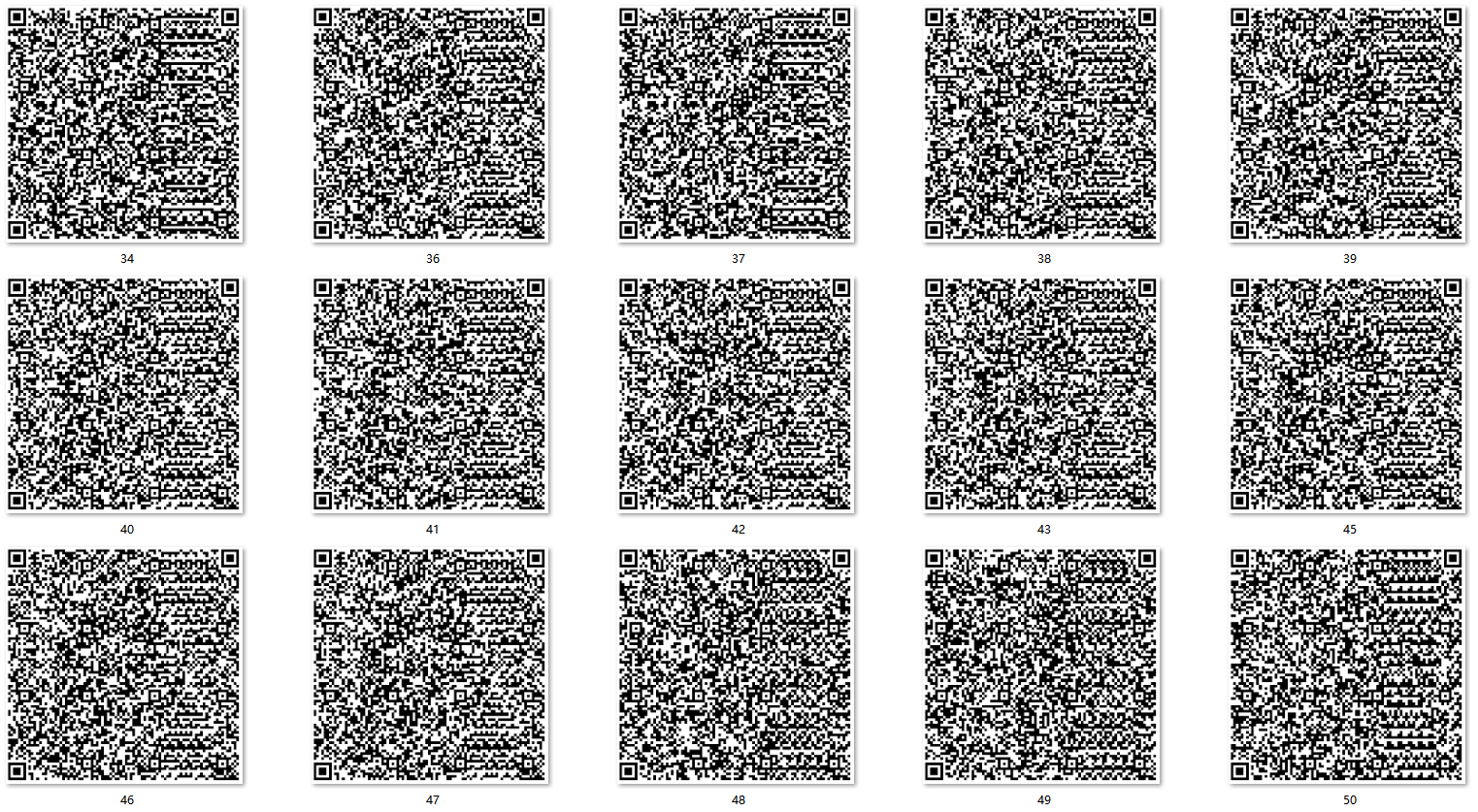
**

**
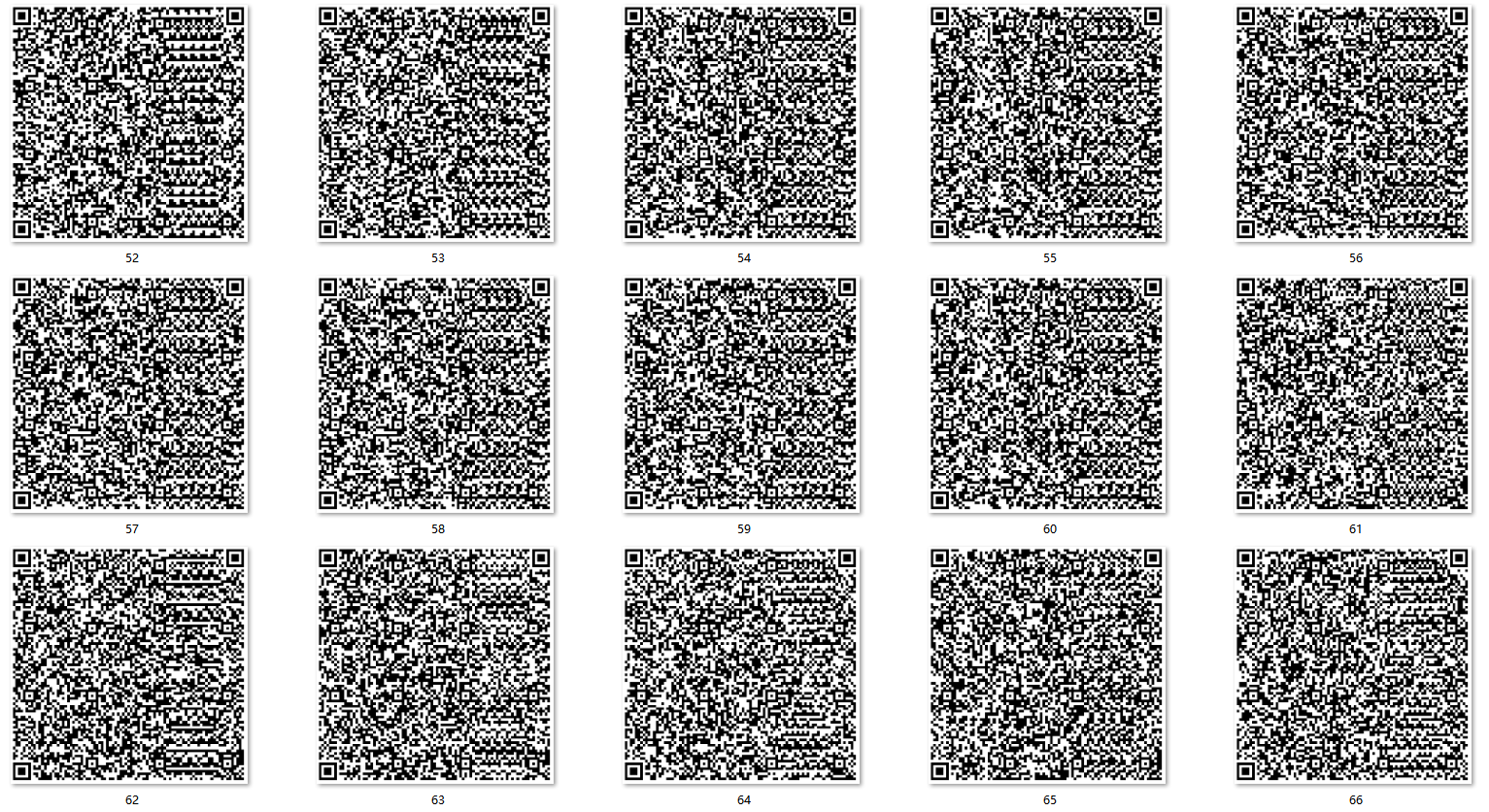
**

**
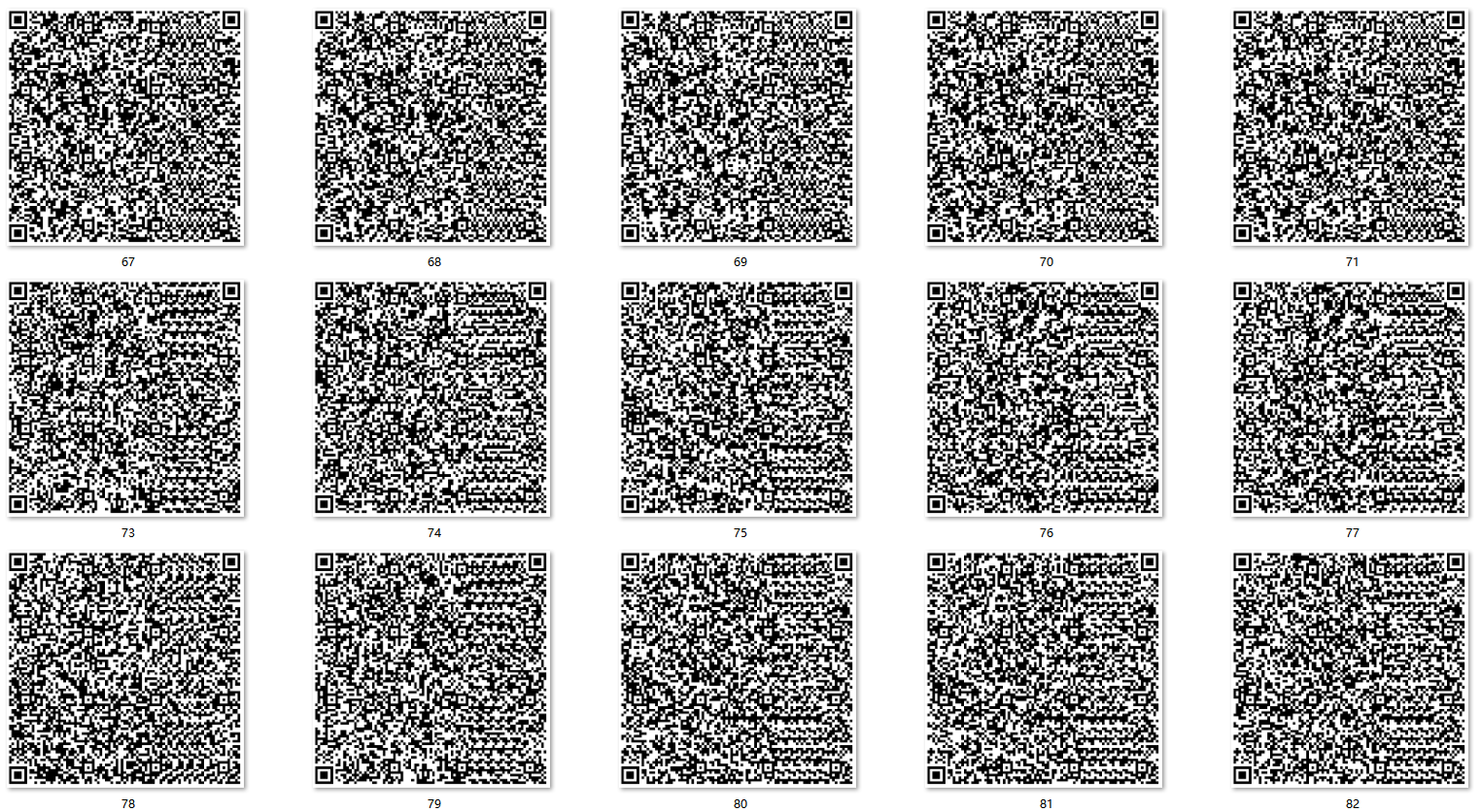
**

**
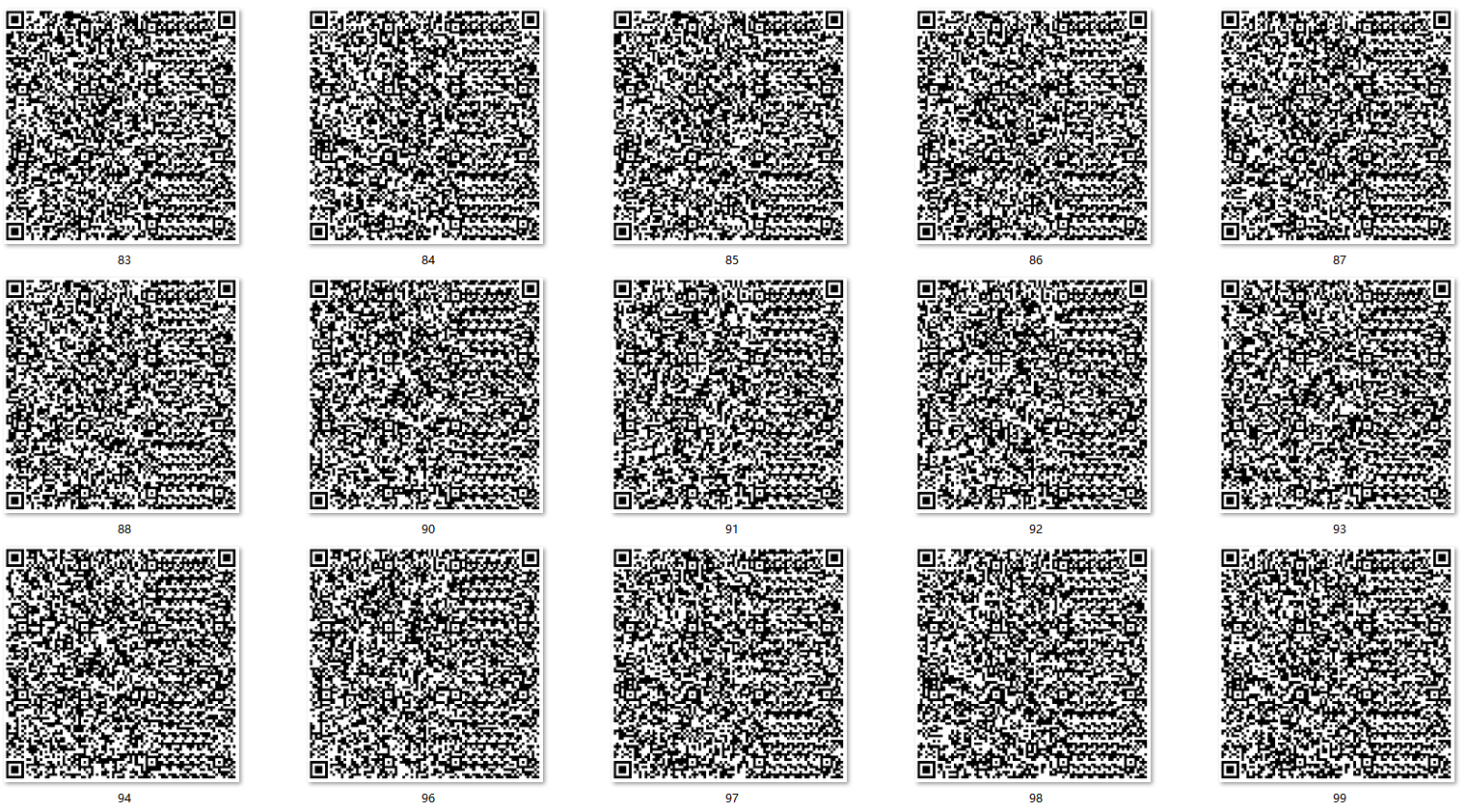
**

**
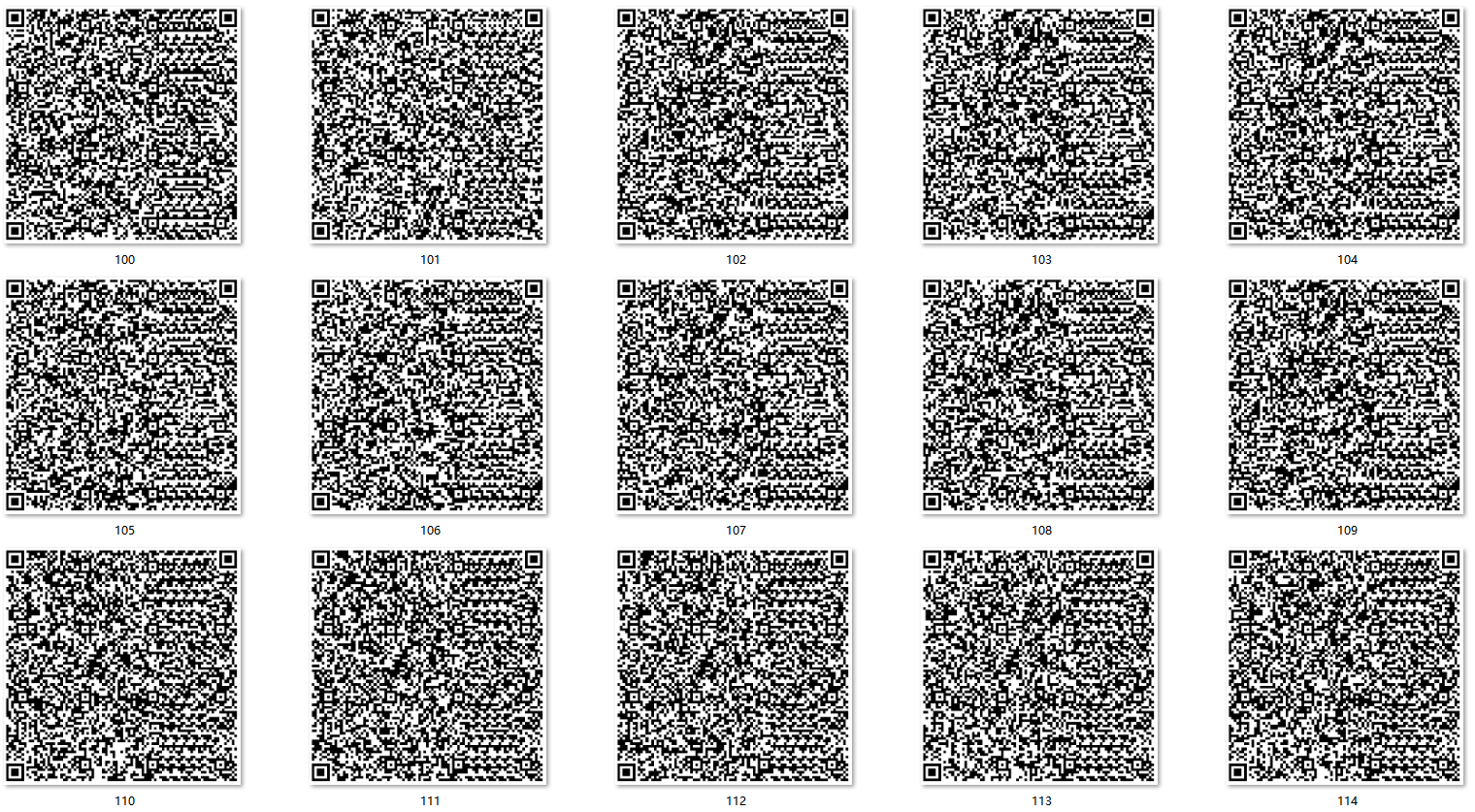
**

**
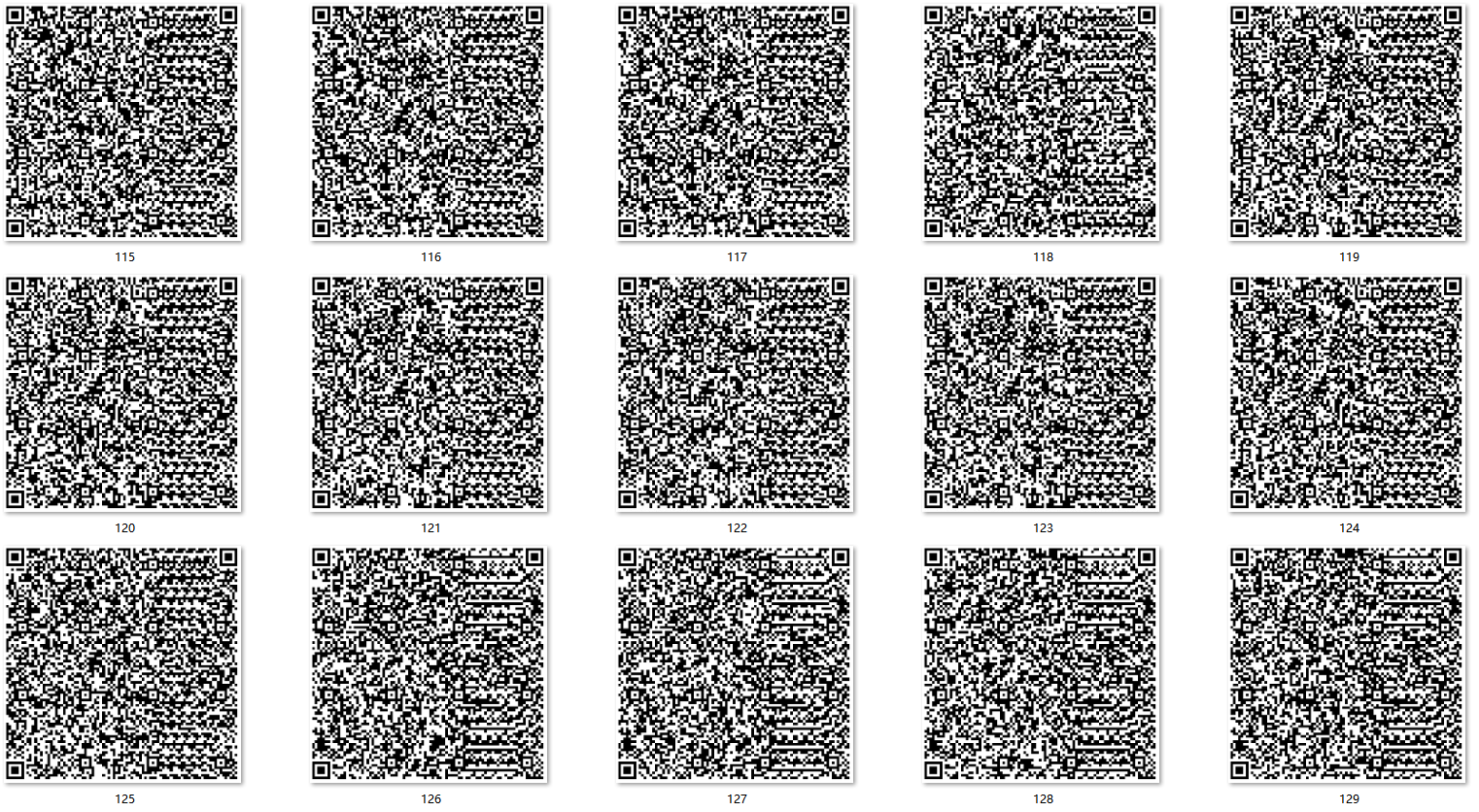
**

**
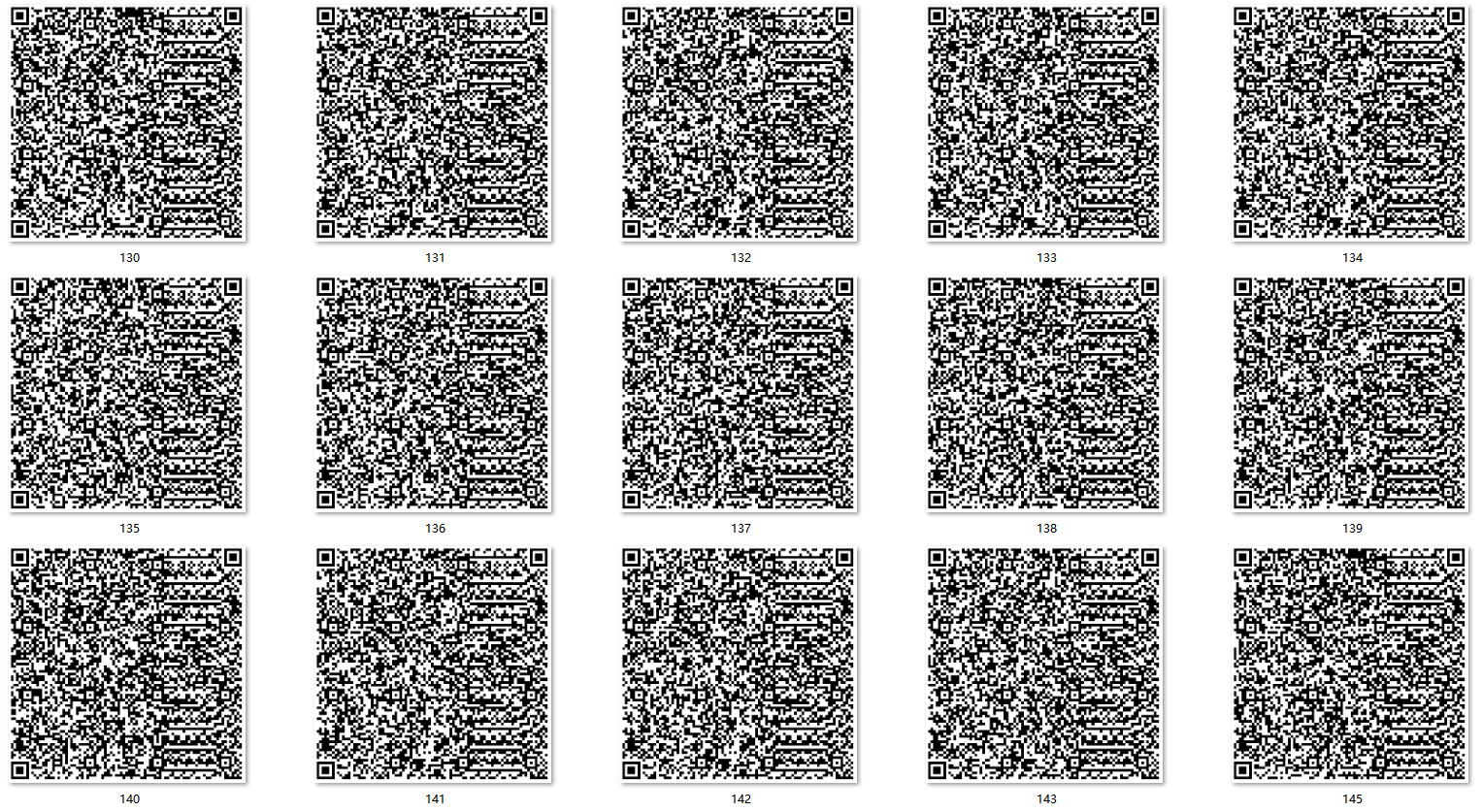
**

**
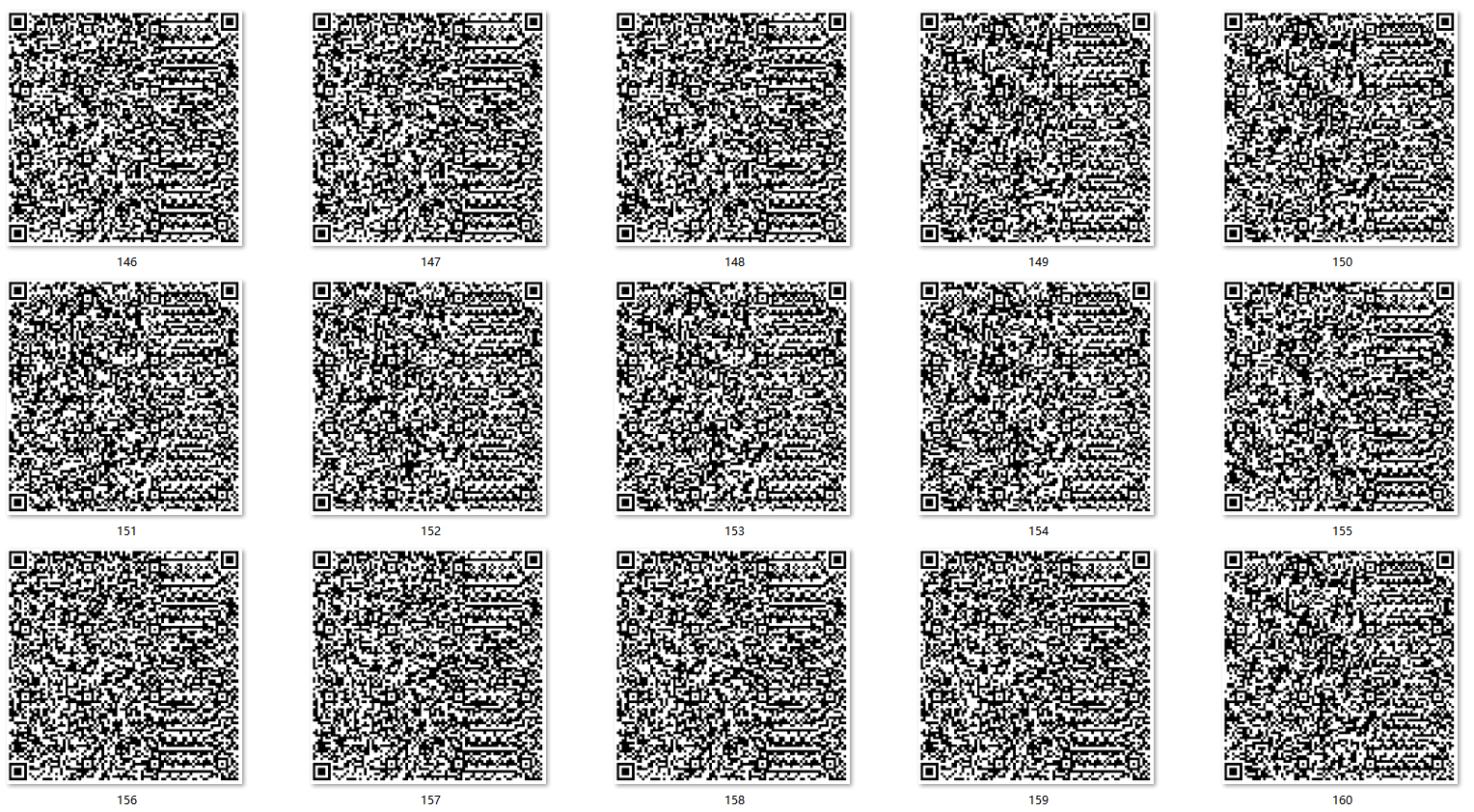
**

**
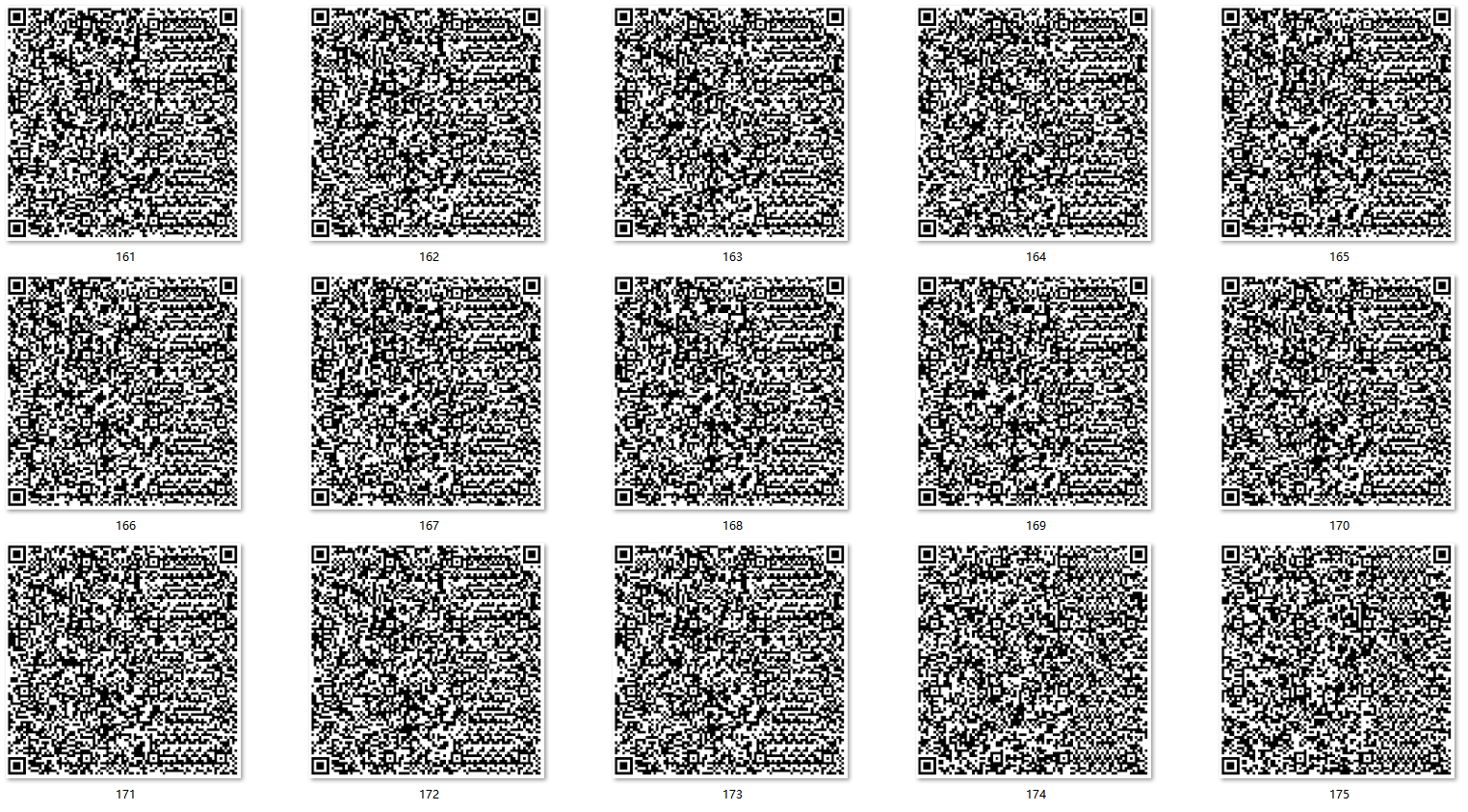
**

**
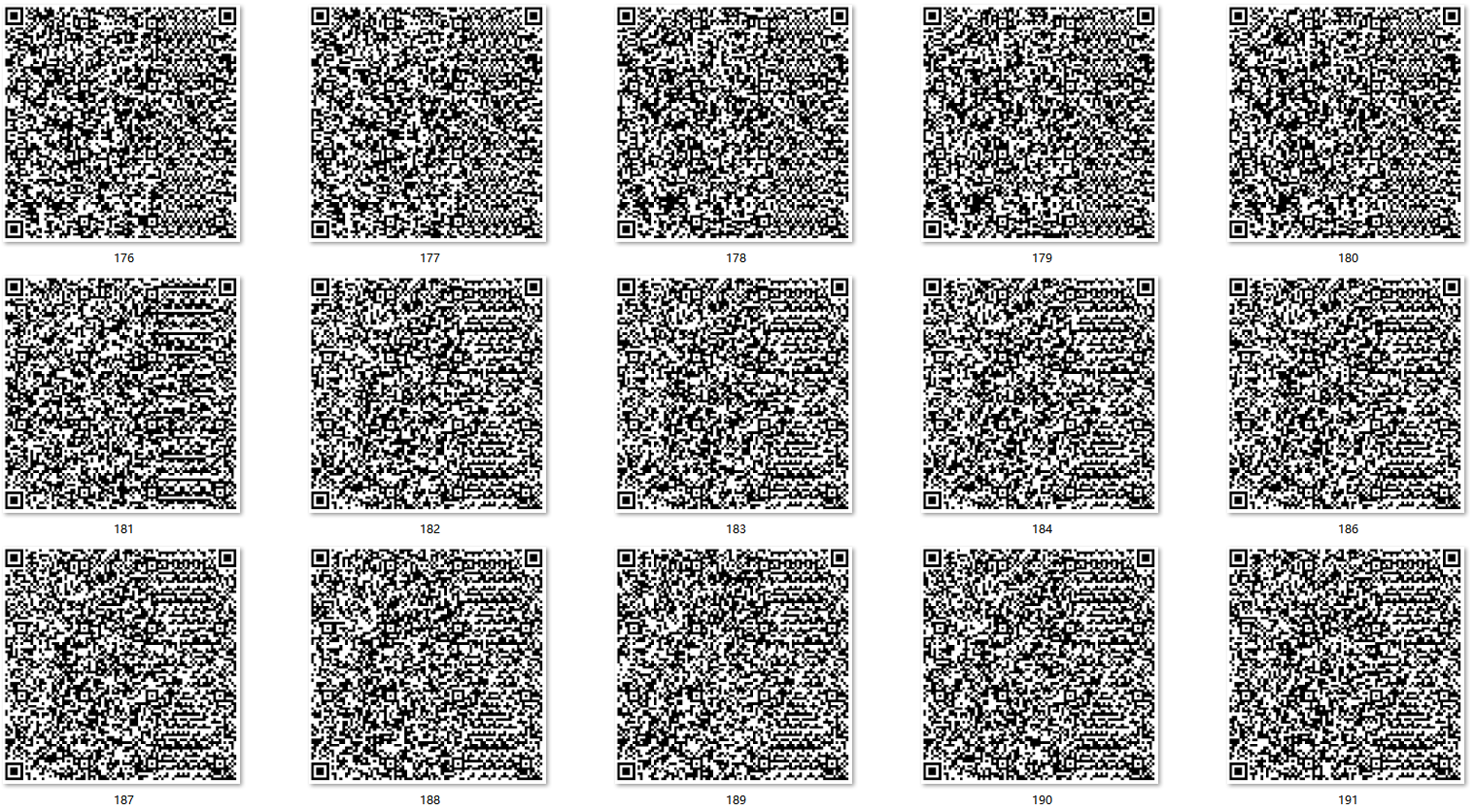
**

**
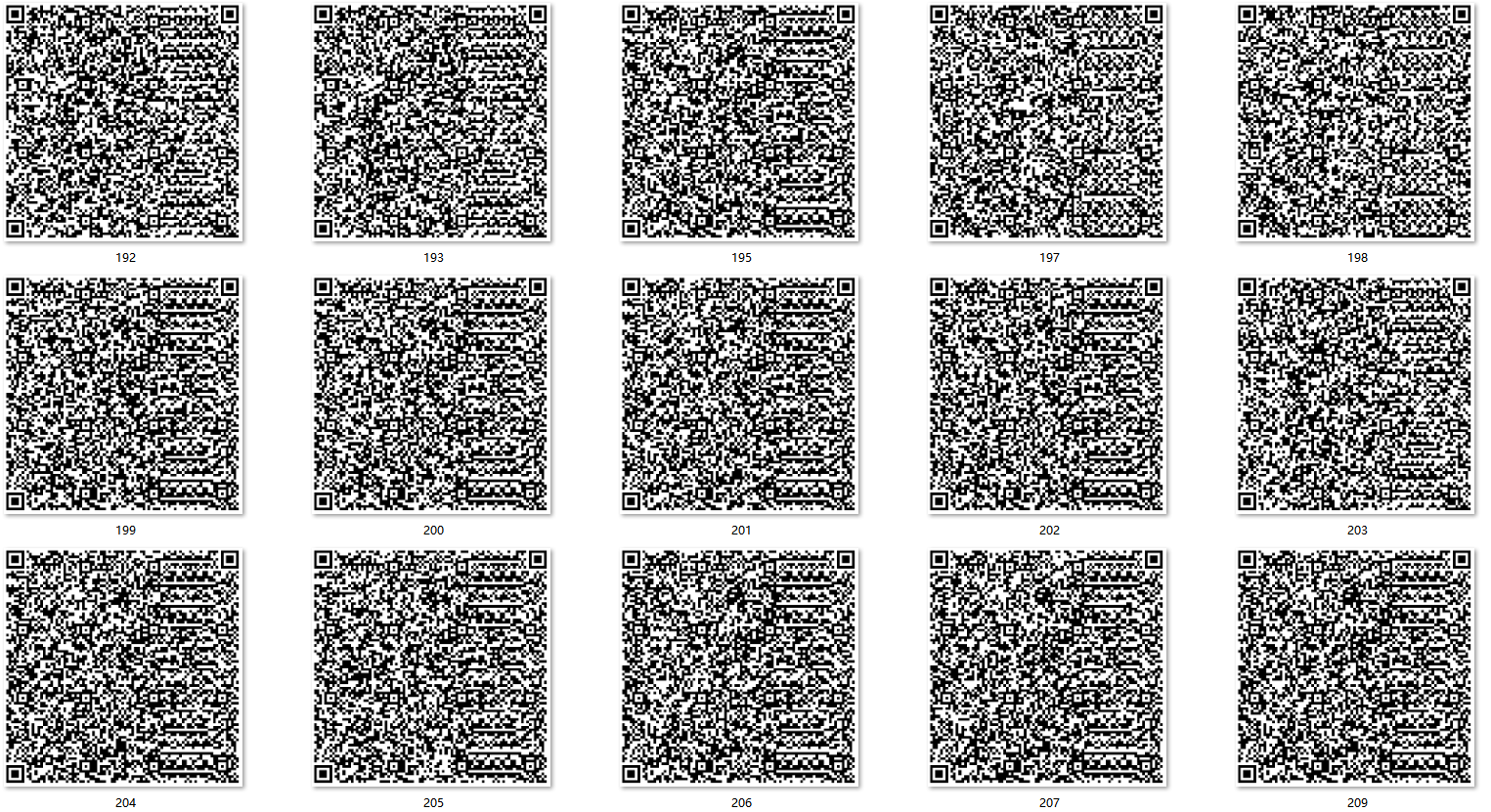
**

**
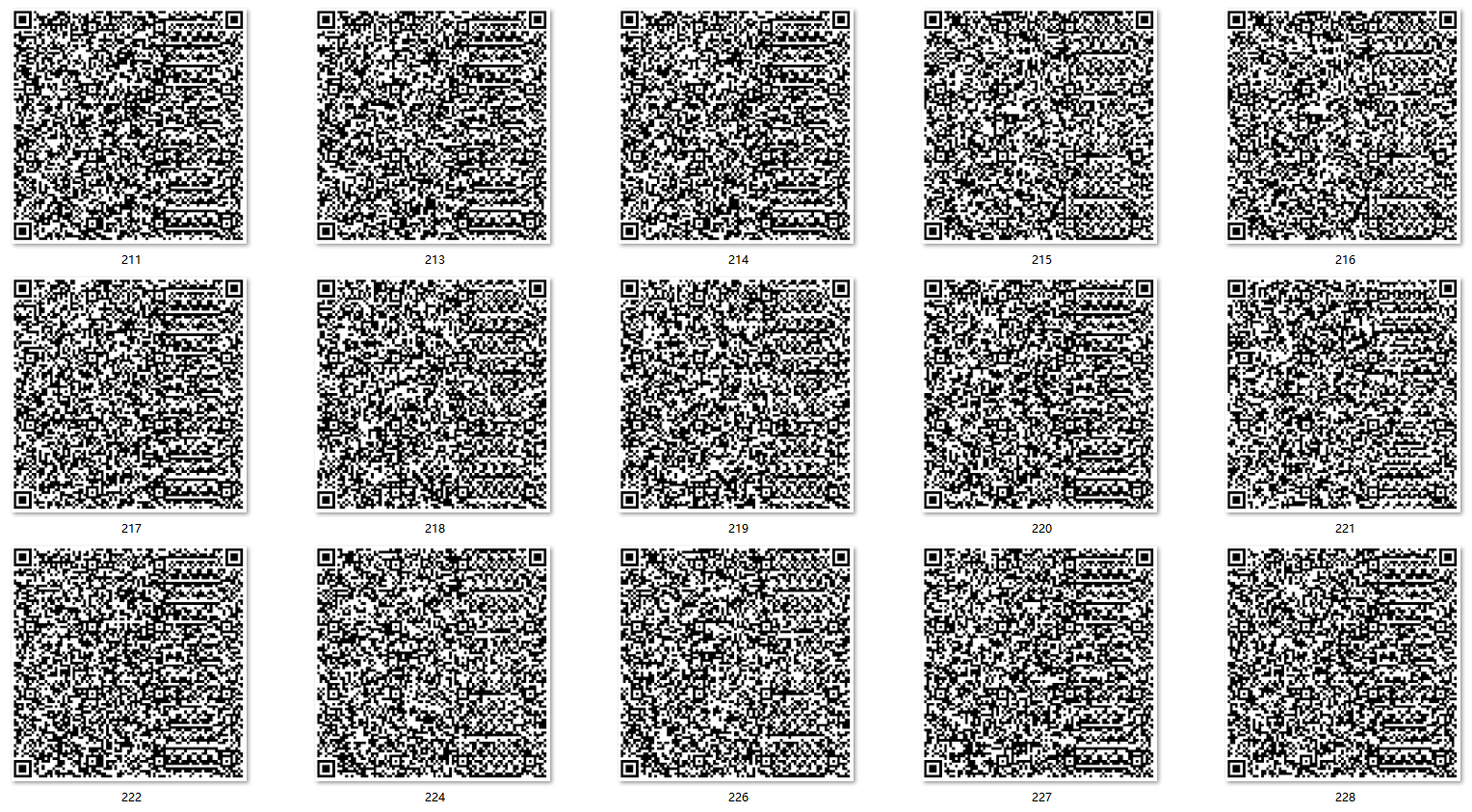
**

**
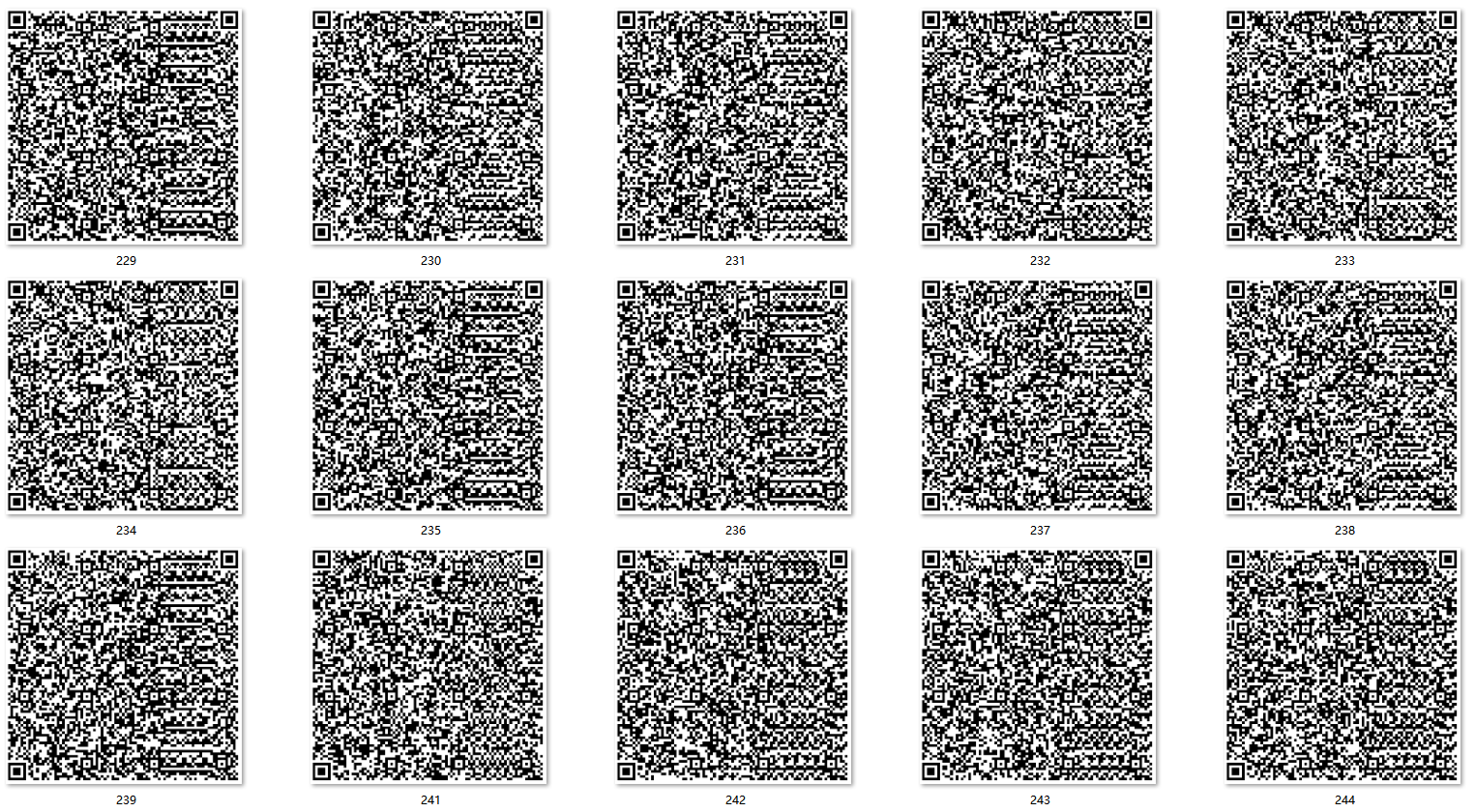
**

**
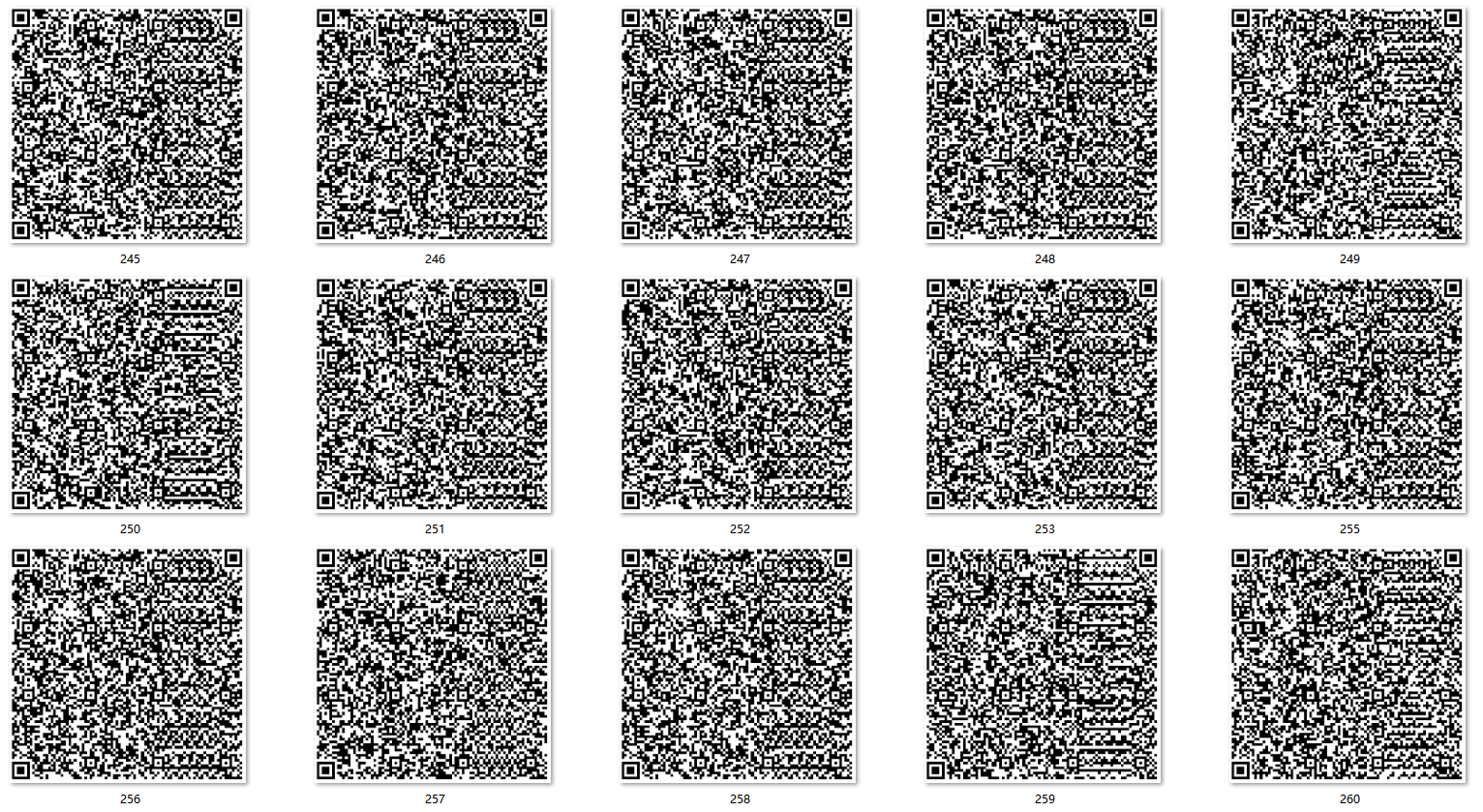
**

**
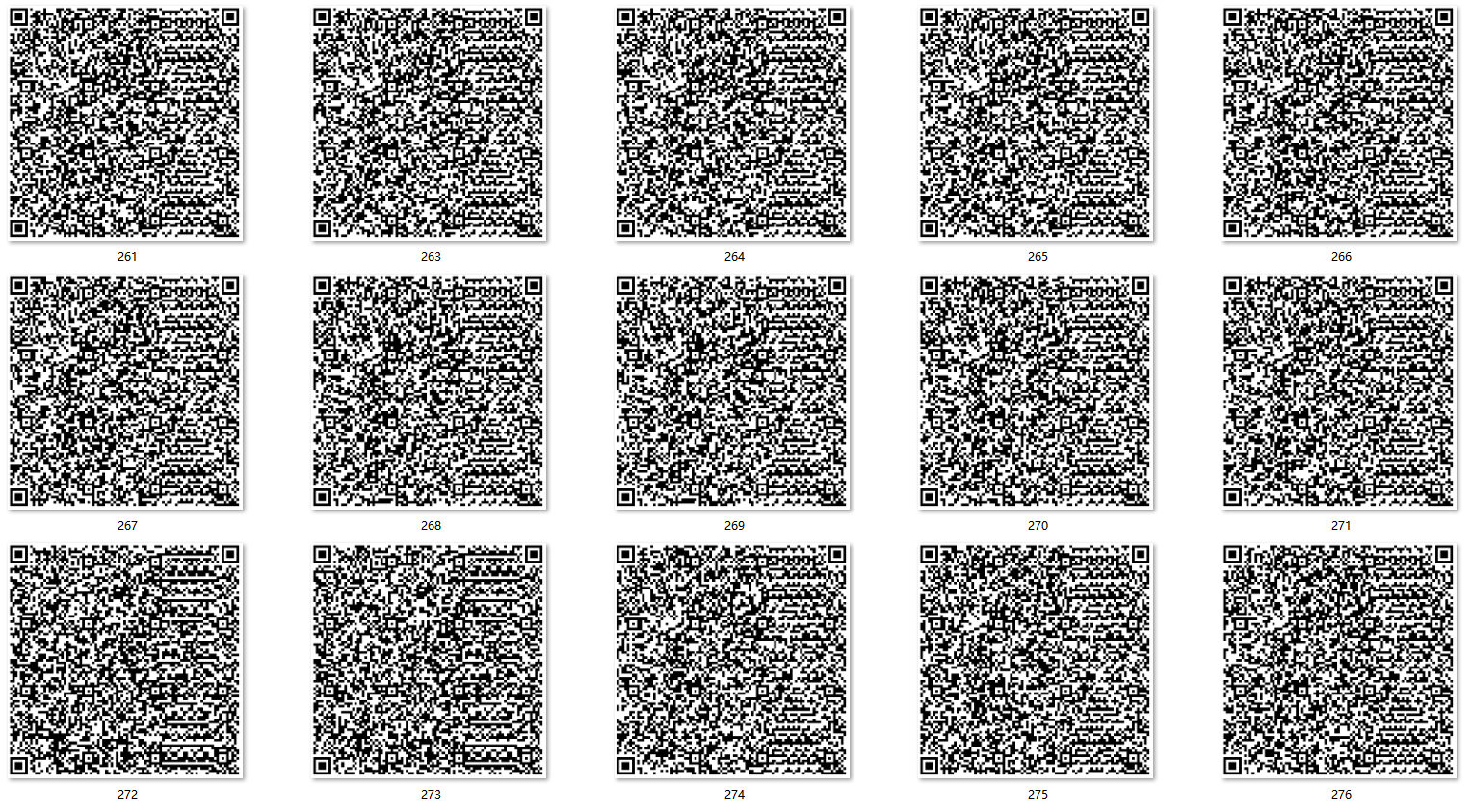
**

**
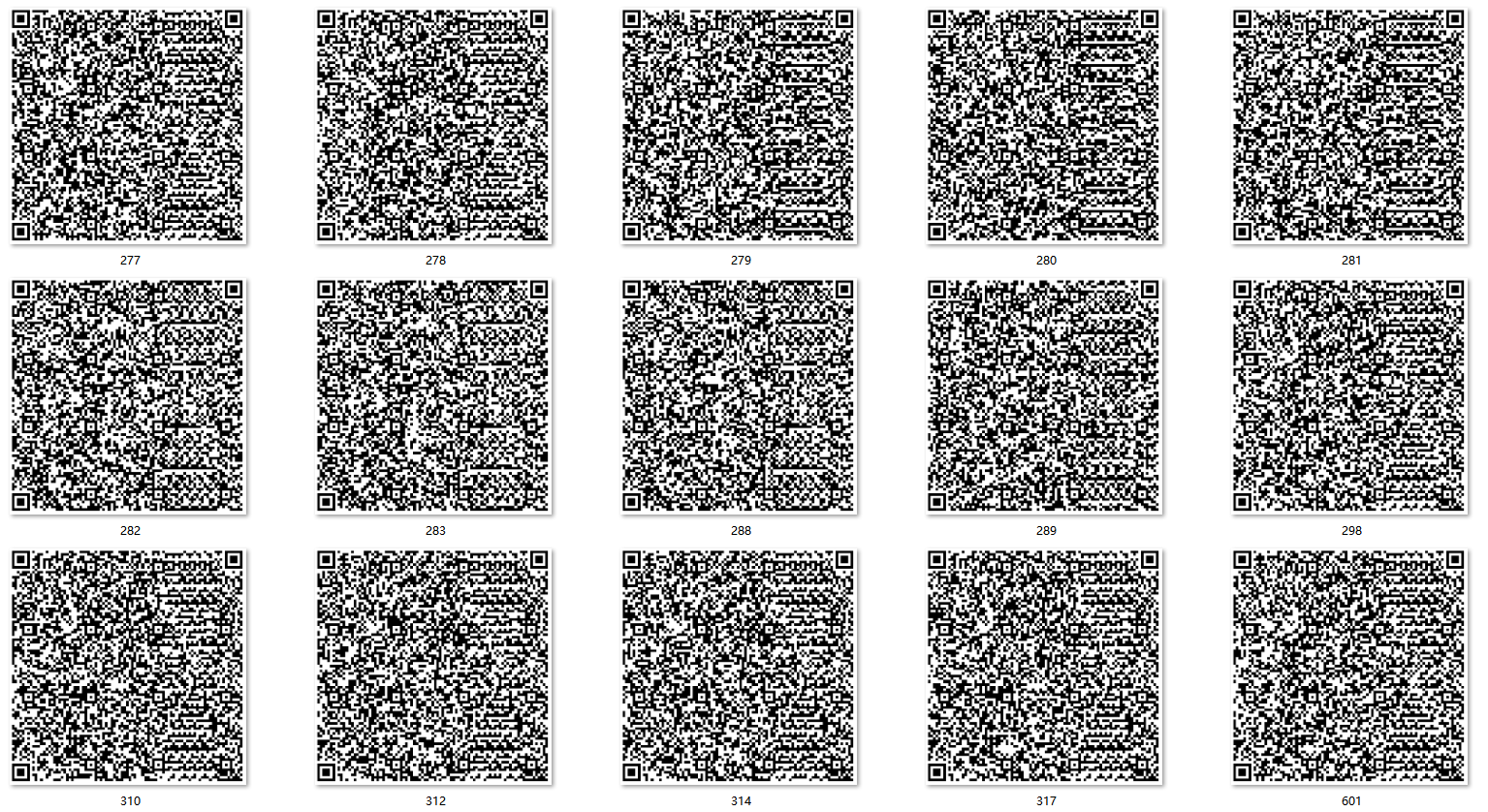
**

**
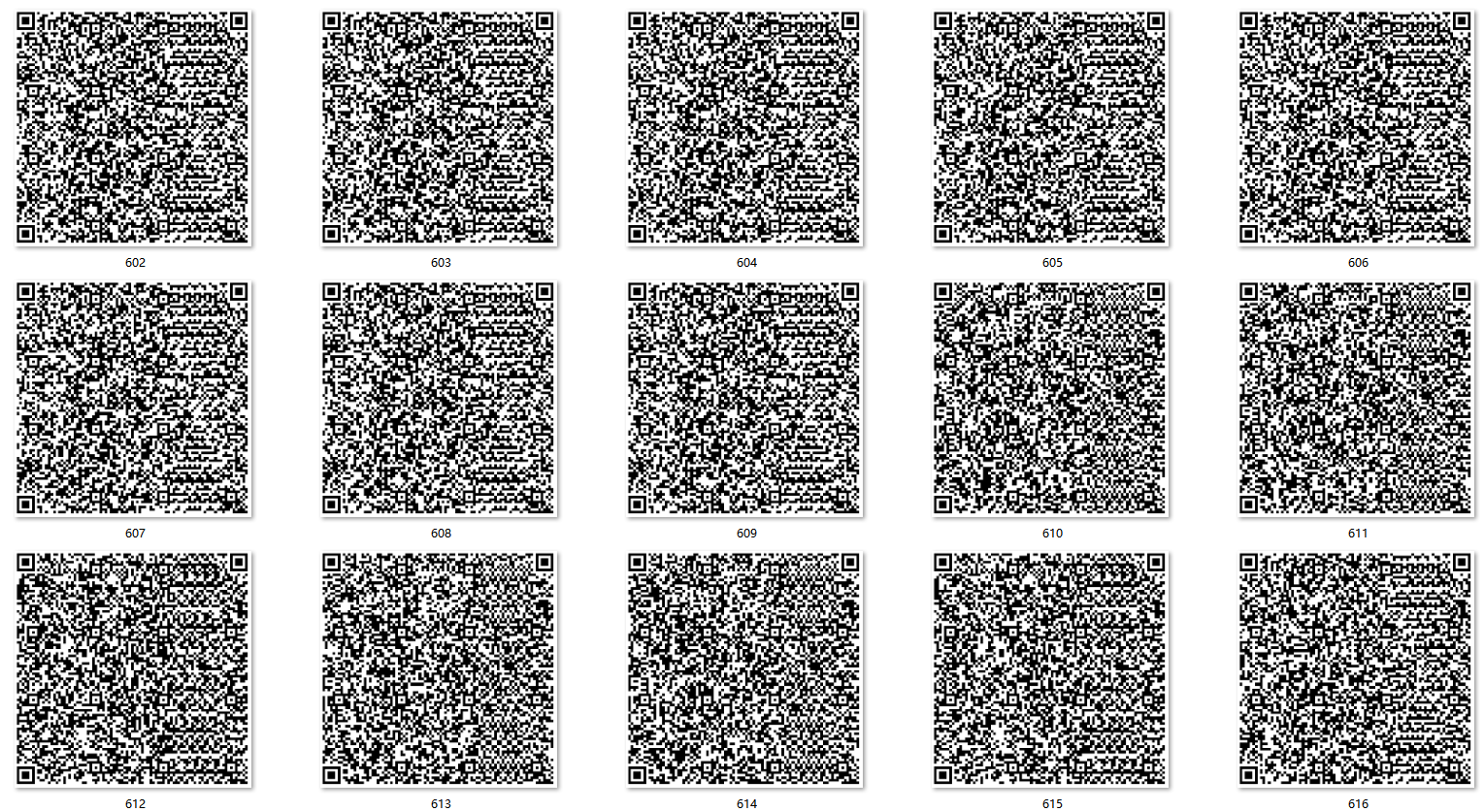
**

**
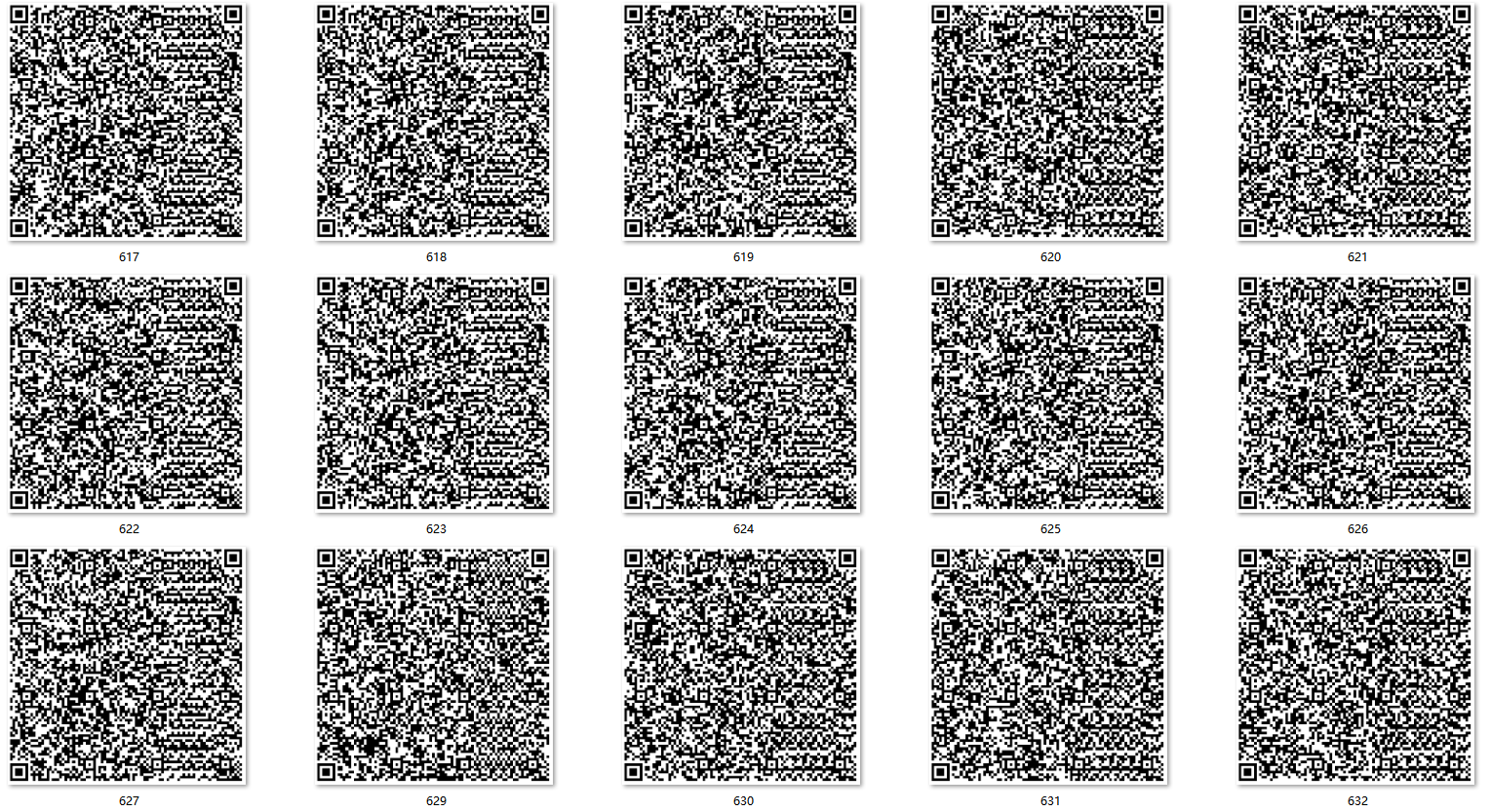
**

**
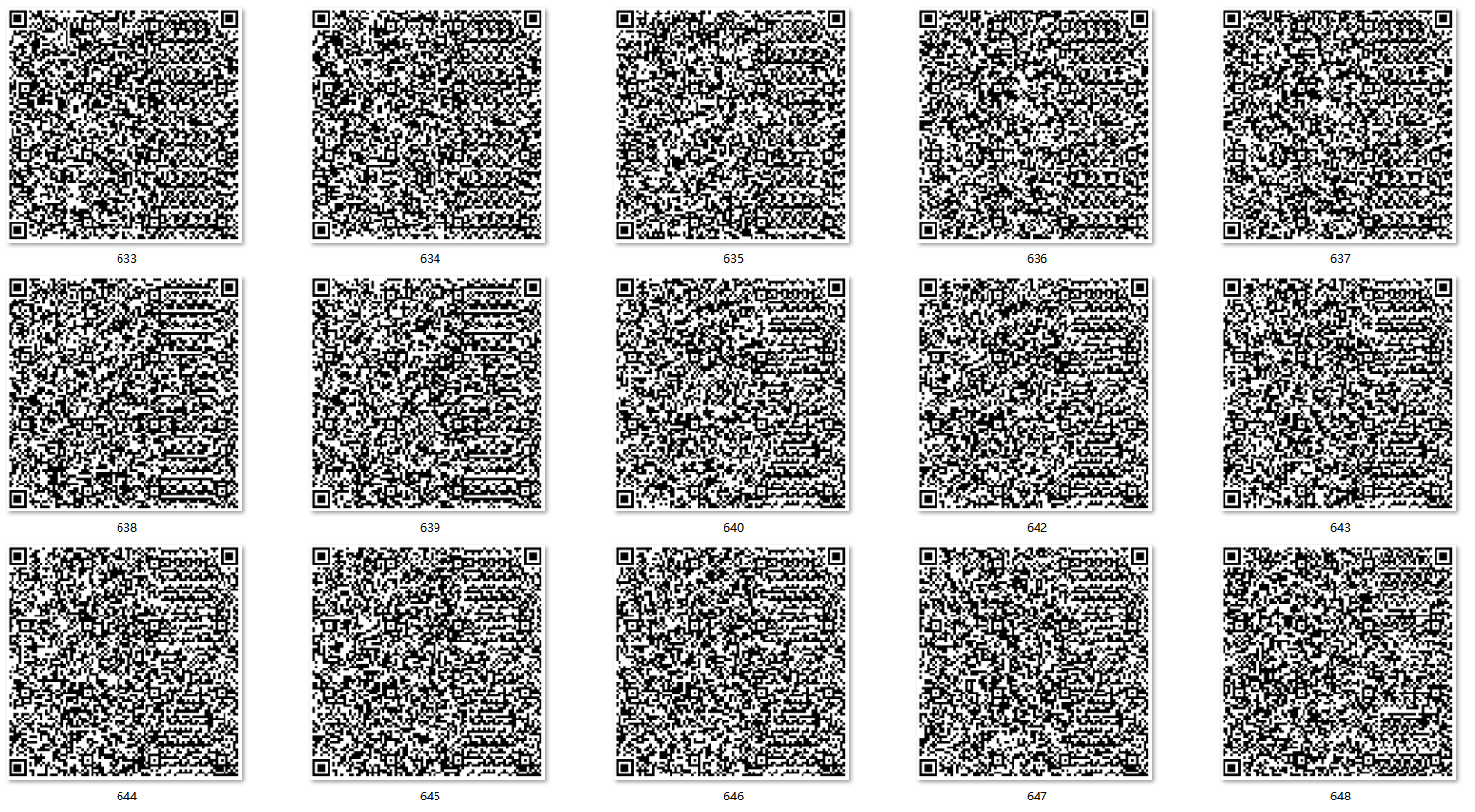
**

**
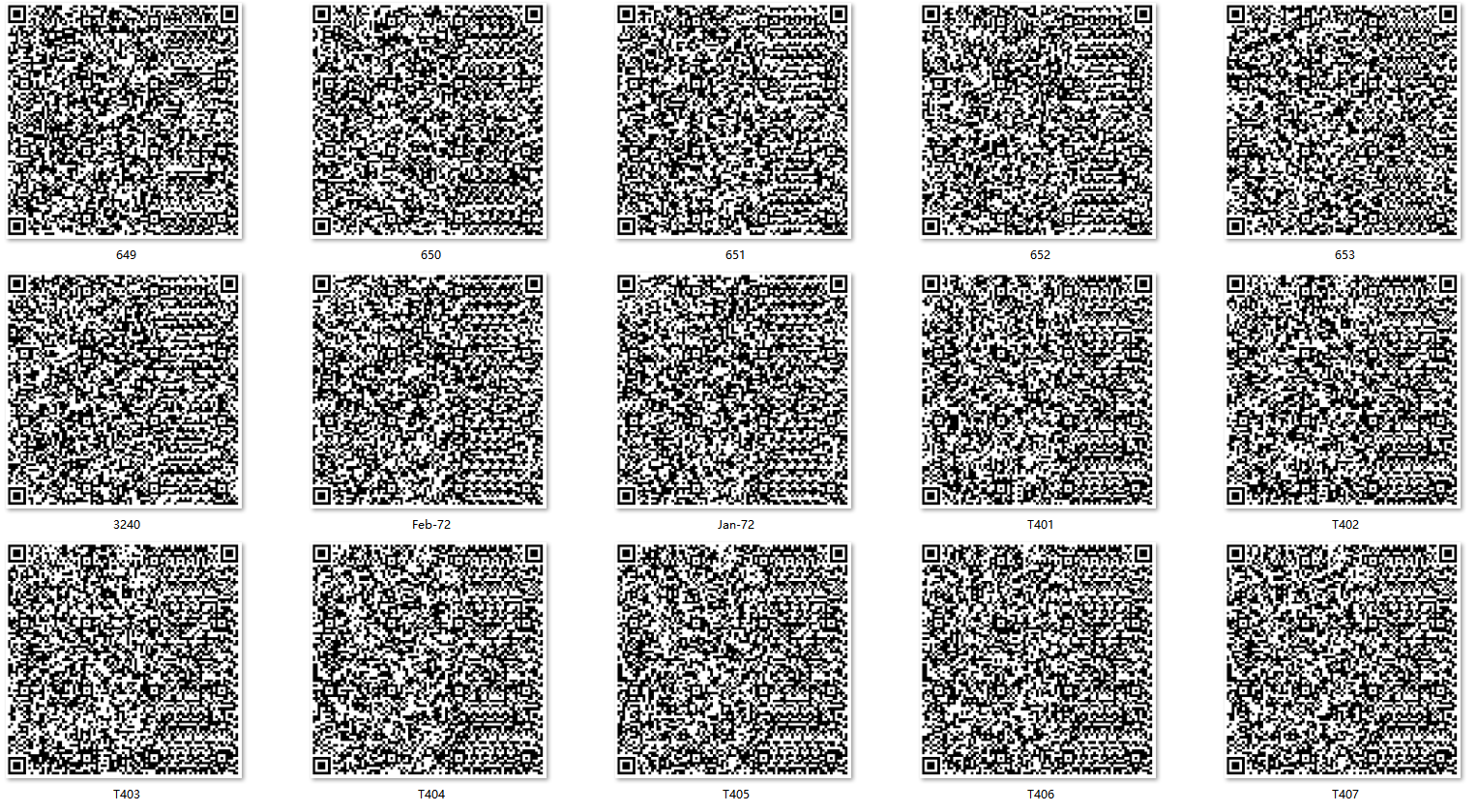
**

**
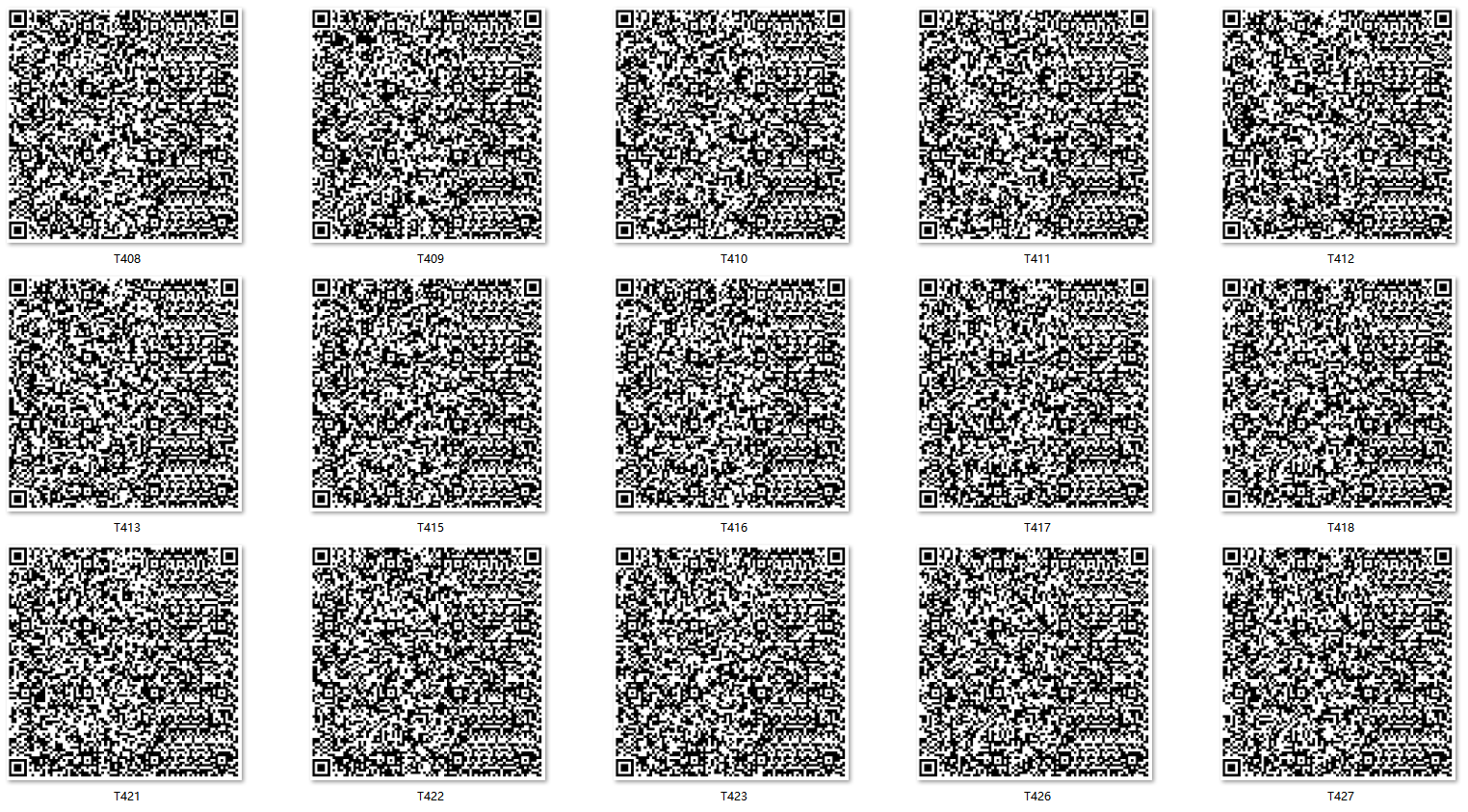
**

**
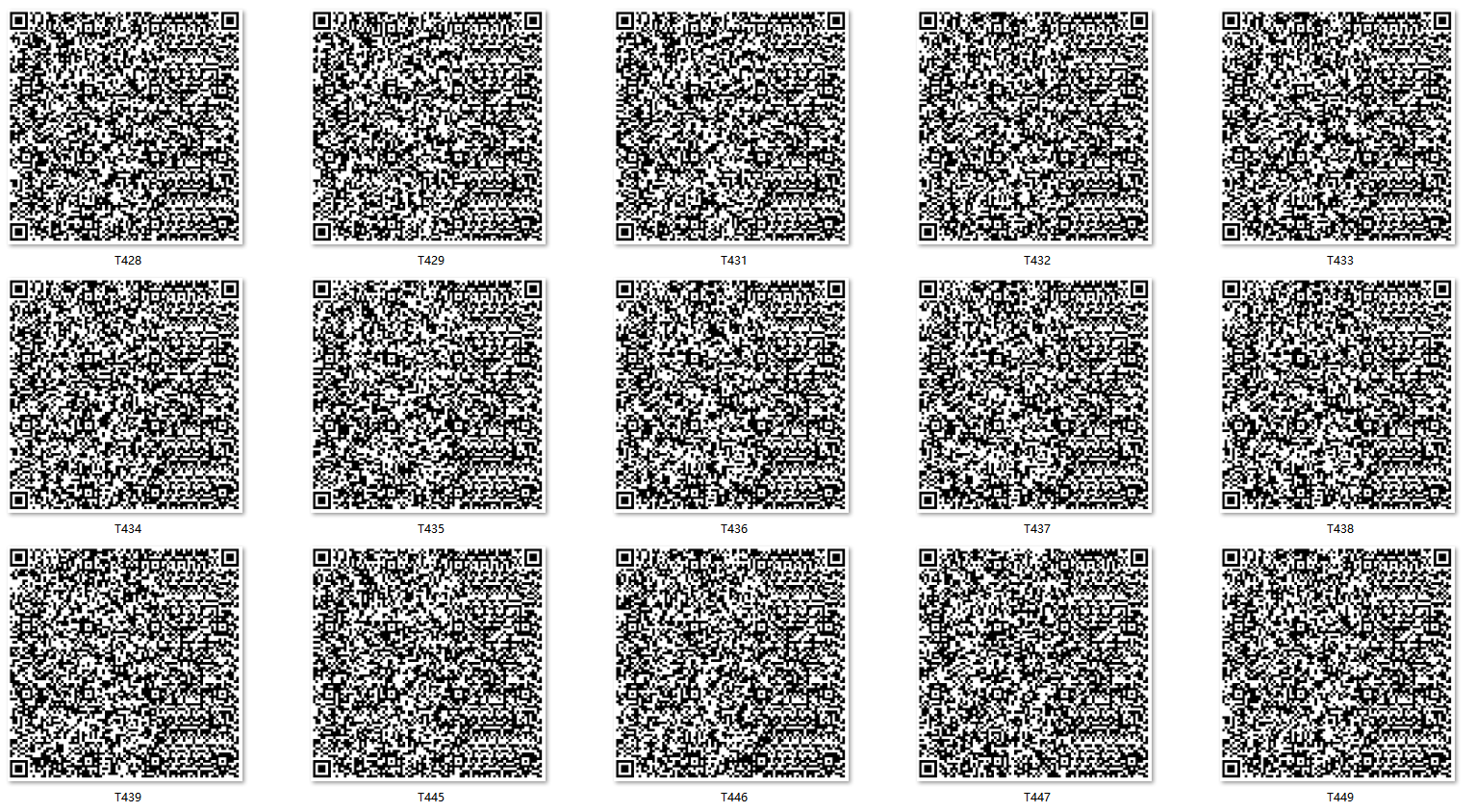
**

**
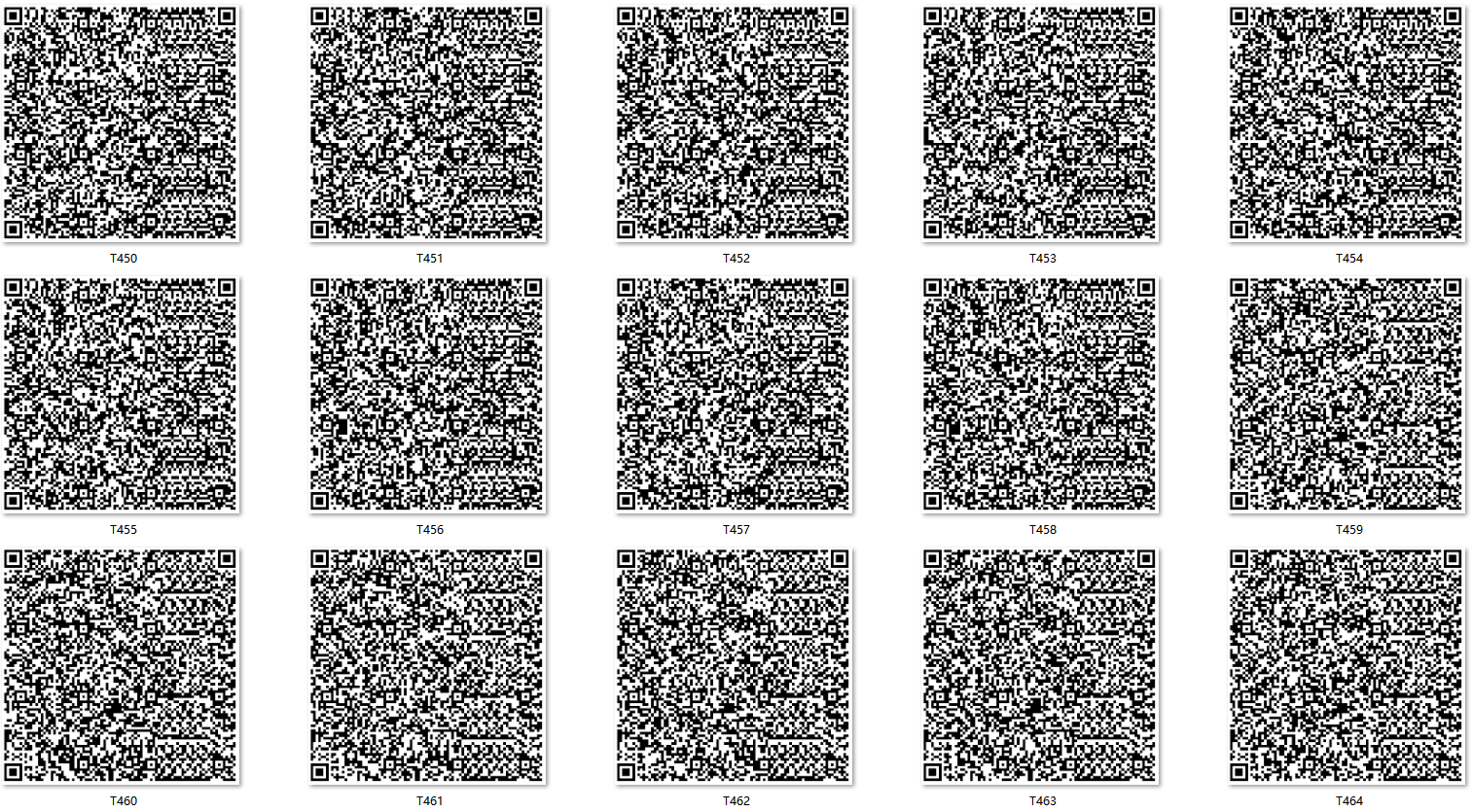
**

**
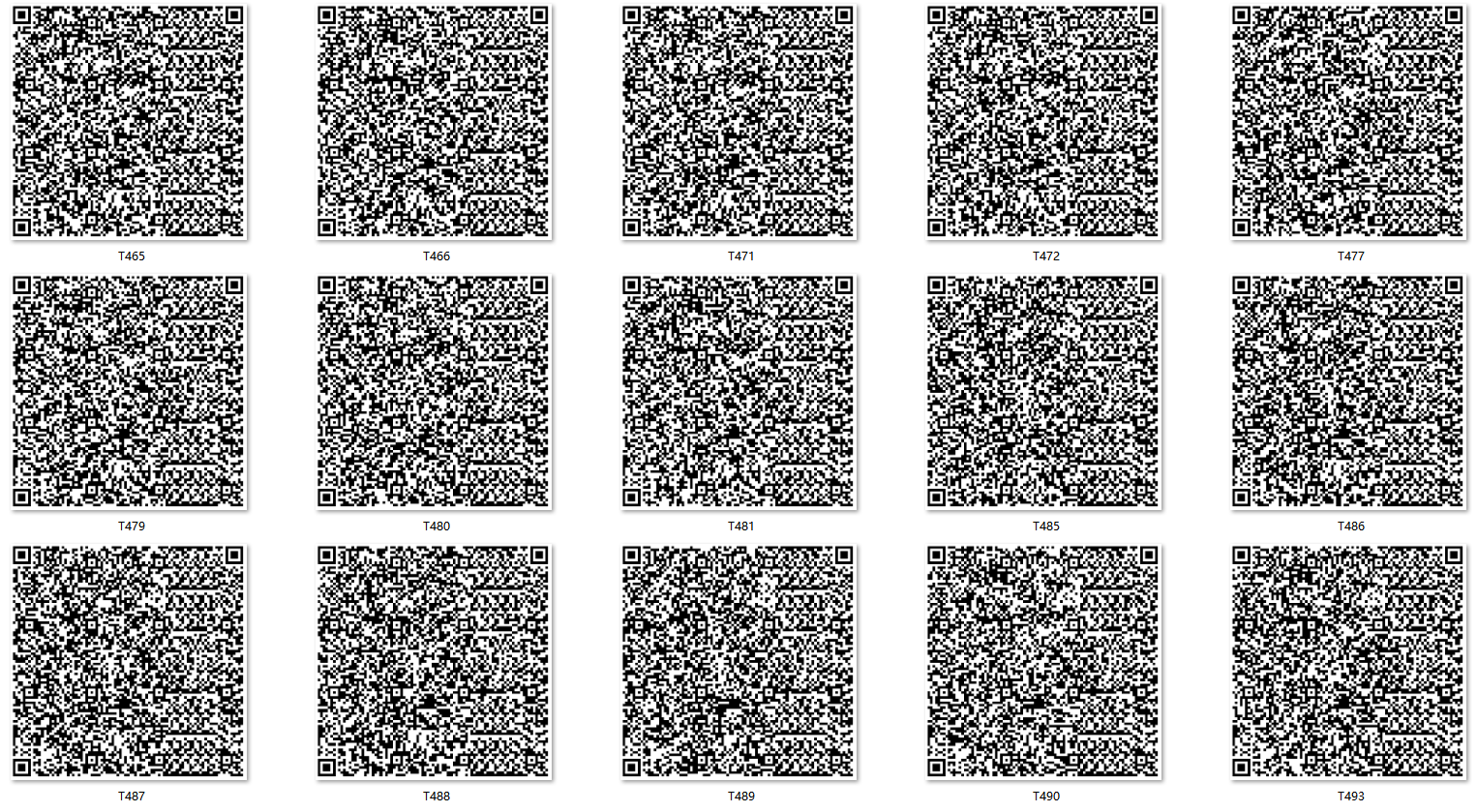
**

**
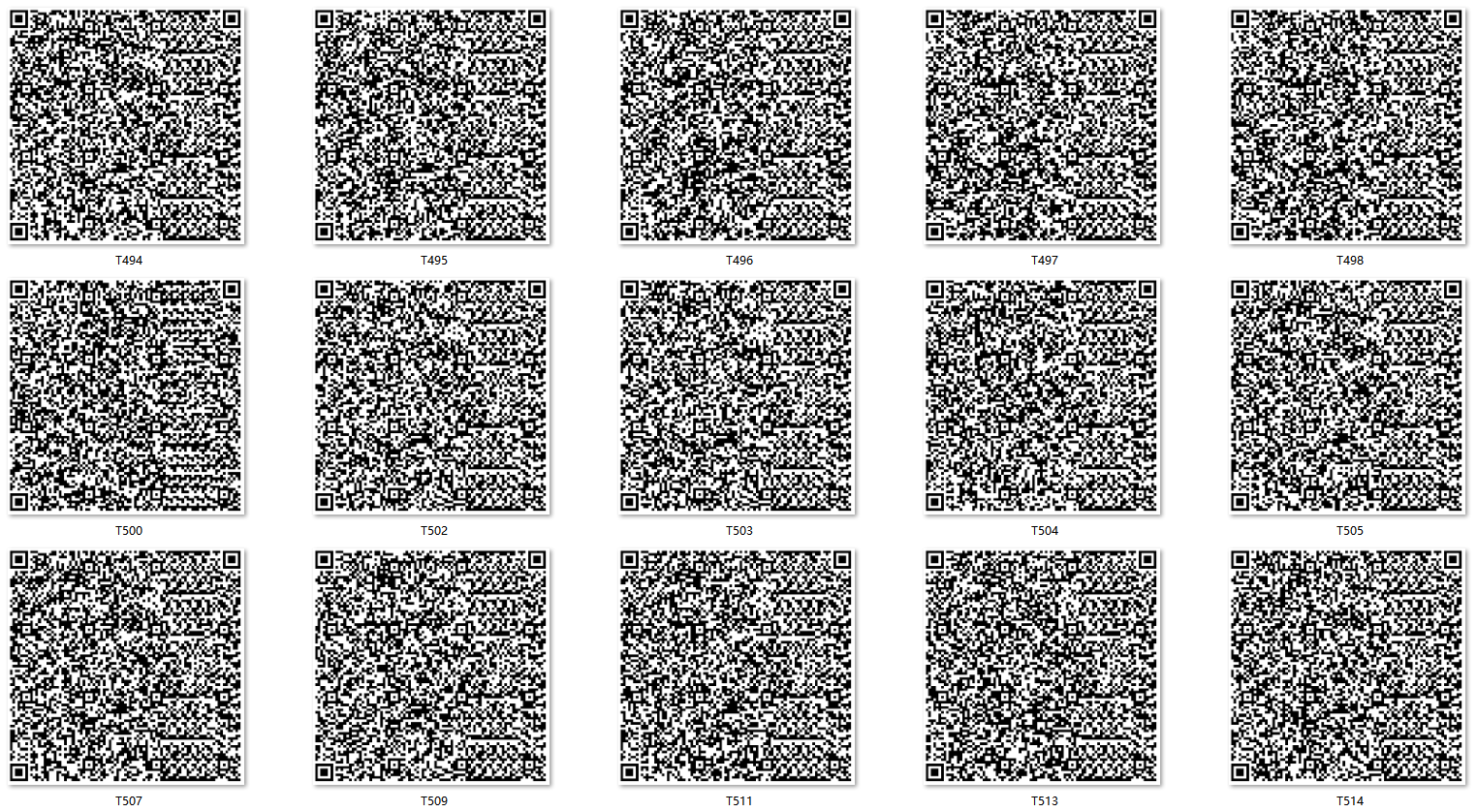
**

**
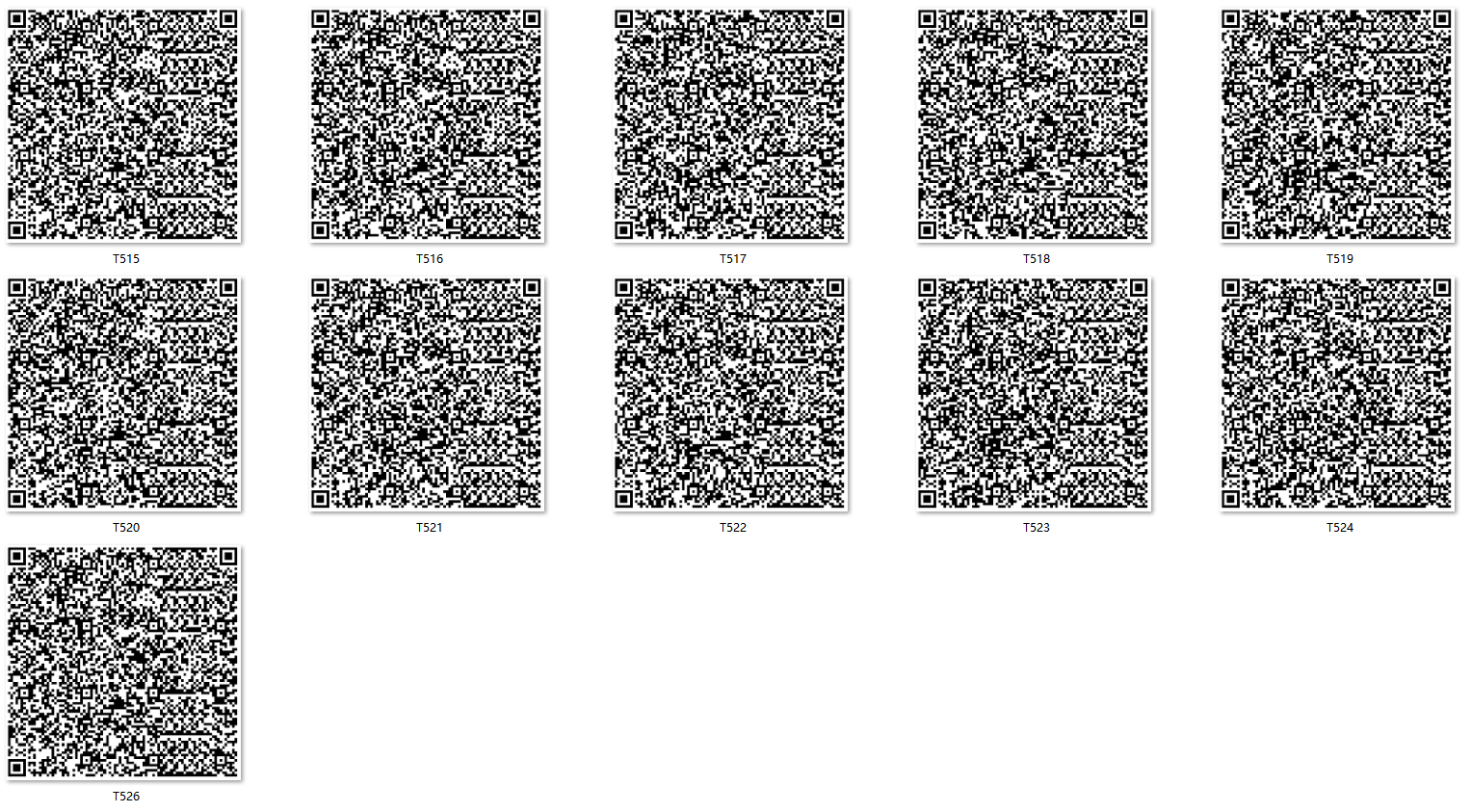
**

**Figure S3.** Information of the GBTS and KASP markers used in this study.
